# Supplementary material for: SCAR-6 elncRNA locus epigenetically regulates PROZ and modulates coagulation and vascular function
Source: EMBO Rep. 2024 Oct 2;25(11):4950–78. doi: 10.1038/s44319-024-00272-w (PMC11549340; doi:10.1038/s44319-024-00272-w)
Supplement: Supplementary file 1 — Appendix file [file 44319_2024_272_MOESM1_ESM.pdf]

SCAR-6 elncRNA locus epigenetically regulates *PROZ* and modulates coagulation and vascular function

Ranjan *et al.*

Short title: *SCAR-6* in coagulation & vascular function

Address for Correspondence:

Sridhar Sivasubbu, PhD., CSIR-Institute of Genomics and Integrative Biology, Delhi, 110025, India. E-Mail: sridhar@igib.in, sridhar.sivasubbu@gmail.com

Vinod Scaria, MBBS, PhD., CSIR-Institute of Genomics and Integrative Biology, Delhi, 110025, India. E-Mail: drvinod@gmail.com,

Table of content:

1. Page 3: Appendix Data

2. Page 4-7: Appendix tables S1, S2, S3, S4

3. Page 8-22: Appendix Figures and Legends S1-S17

Appendix Data

CRISPR-CAS9 mediated *scar-6* mutant generation

We designed the sgRNA targeting the 1st and 2nd exon of scar-6 lncRNA and checked the off targets of these sgRNA with 4-bp mismatch using Cas-OFFinder web tool (http://www.rgenome.net/cas-offinder/). sgRNAs showing no off- target with 4-bp mismatch and was selected. sgRNA IVT was generated as previously described in Varshney et al. 2015. The sgRNA an Cas9 RNP complex was injected into single cell zebrafish. The injected F_0 animals were then screened for any cardiovascular phenotype such as heart defect, vascular abnormality or haemorrhage. The sgRNA targeting the 5' Region of the scar-6 did not show any cardiovascular related phenotype whereas the sgRNA targeting 3' region showed ~30% animals with haemorrhage phenotype. We next followed these F_0 animals and they were grown to adulthood and then mutation screening for hetrozygous using heteroduplex mobility shift assay (HMA) on fin-clipped DNA was performed. In this assay, two PCR strands with different sequences (WT and mutant) are allowed to anneal, forming heteroduplexes. The mobility of these heteroduplex structures through the gel is influenced by their size, shape, and charge. By assessing the mobility shift of the heteroduplexes compared to the wildtype PCR template strands we can screen for the hetrozygous animals. We observed multiple bands in the PAGE gel compared to wild type, conforming mutation in the zebrafish. We observed multiple indels at the target loci in the $F_{0.}$ The F_0 zebrafish, positive for hetrozygous mutation was outcrossed with wild-type *gib004Tg*(*fli1: EGFP; gata1a: dsRed*) animals, and similarly, F_1 hetrozygous mutation positive animals were again outcrossed with wild-type *gib004Tg*(*fli1: EGFP; gata1a: dsRed*) animals to get a stable mutant line (Fig EV4F). F_2 mutant were genotyped and heterozygous mutant zebrafish animals were identified. We generated a stable mutant line with 12 bp deletion at the target loci of *scar-6* which were named *scar-6*$^{gib007\Delta12.}$ (Fig 3B)

Appendix Table S1: Details of prioritized 10 *scar* lncRNA genes

Name	**ZFLNC ID**	**Neighbouring protein coding gene**
scar-1	ZFLNCG01330	*ghrhra; adcyap1r1a; Scn12aa*
scar-2	ZFLNCG05739	*prlh; rpe; ackr3a; tyw5; maip1*
scar-3	ZFLNCG09043	*Apoa.4; Apoeb.2; Apoc*
scar-4	ZFLNCG09044	*Apoa.4; Apoeb.2; Apoc*
scar-5	ZFLNCG11113	*selp; sele*
scar-6	ZFLNCG00003	*f7i; f10; prozb; pcid2; cul4a*
scar-7	ZFLNCG00988	*myh7l; myh7*
scar-8	ZFLNCG00989	*myh7l; myh7*
scar-9	ZFLNCG13000	*sulf1; csrnp1b*
scar-10	ZFLNCG07667	*pax2a; cuedc2; hif1an; wnt8b; scdp; dnajb12a; trmt2b*

Appendix Table S2: Publicly available datasets used in this study after reanalysis.

**Sno.**	**Public data repository**	**Comment**
1	GSE32900	Development stages of zebrafish
2	GSE134055	Tissues of zebrafish
3	PRJNA504385	Zebrafish Endothelial cell
4	GSE133437	CTCF ChIP-seq

Appendix Table S3 : Genotypic percentage of animals showing haemorrhage phenotype in in-crossed scar-6 mutants.

	No-phenotype		Phenotype	
	N	%	N	%
Total	285	100.0		
WT	69	24.2	0	0
Heterozygous	132	46.3	0	0
Homozygous	14	4.9	70	24.6

Appendix Table S4: Primer details.

S. No	Name	Sequence (5'-3')
1	3'RACE scar-6 F	GCCAAAAGCCTGTAGCCATT
2	3'RACE nested scar-6 F	AGTCCTCGAGACACCACTGACCTCCATAGT
3	T7+scar-6 full length R	TAATACGACTCACTATAGGCGCAAGGGGAAATACGGCG
4	scar-6 full length F	CGAAGCATTGATGAACCATTC
5	T7+scar-6 full length F	TAATACGACTCACTATAGGGCGAAGCATTGATGAACCATTC
6	scar-6 full length R	CGCAAGGGGAAATACGGCG
7	RT_zff10F	AGAAGAATGTGGTCTGCTCG
8	RT_zff10R	ATGCTGTCGGTCTGGTTGTT
9	RT_zfprozbF	GGAGACCAGTGCAGATCTAA
10	RT_zfprozbR	CGTCGCTGTTGAGTGTGTAT
11	zf actinb F	AA TTGCTTCCGAGGCGcgctggagctaatcgatga
12	zf actinb R	GGTGGCTCCAACTCGgtgaccatgtggagtcagctt
13	human_scar-6-F	CTGCCCTCCGCGCAGCATGGA
14	human_scar-6-R	GGATCGACAGGTCCATGAAAAC
15	scar-6 exon2 F	TTATTGGTGTCTTGTGCCGC
16	scar-6 exon2 R	AAGGGGAAATACGGCGTCTA
17	PAR1(f2r) F	CCC CCG GCT AAA AAG ACT TA
18	PAR1(f2r) R	GGCTCCGTATATCCAGTTGT
19	PAR2a F (f2rl1.1)	ATTGAACAGCAAGCTCACGC

**20**	PAR2a R	CAGTACCTCTGGACGCTAAT
**21**	PAR2b F (f2rl1.2)	CATCTGGACGCCTCTAAAGA
**22**	PAR2b R	TGATGATGTTGACGTCGTGG
**23**	PAR3 F (f2rl2)	TCATCAATCACACCGCTGGA
**24**	PAR3 R	AGCTTGCAAGCTAGTTCACC
**25**	T7+f10 F	TAATACGACTCACTATAGGGTCC TGA ACT CTG CGA GAA TG
**26**	T3+f10 R	GCAATTAACCCTCACTAAAGGGAAATTGCTGGACTCGATGC
**27**	T7+prozb F	TAATACGACTCACTATAGGGAAA TCC CAG TGT CCA TCT GC
**28**	T3+prozb R	GCAATTAACCCTCACTAAAGGGCCTGTCAGAAAGGCTGTTT
**29**	f10 F	TCC TGA ACT CTG CGA GAA TG
**30**	f10 R	GAAATTGCTGGACTCGATGC
**31**	prozb F	AAATCCCAGTGTCCATCTGC
**32**	prozb R	GCCTGTCAGAAAGGCTGTTT
**33**	CTCF_RT_six 6a F	AATGACCGCTGACAATAGCG
**34**	CTCF_RT_six 6a R	GAGCAGGGTTAAACACTGAC
**35**	CTCF_RT_sca r-6 F	AATACATCATCCCCGCGT
**36**	CTCF_RT_sca r-6 R	CGGTTAGTTTCTGCAGGATG
**37**	CTCF_RT_f7 F	CTACAGTGTGTATGCGGTGC
**38**	CTCF_RT_f7 R	GTATCCGGCGCAGAACAT
**39**	vcam1b F zf	GATGCTGGAACTTACCAGTG
**40**	vcam1b R zf	CTTGACTGTGGACTTGCTAC
**41**	vcam1aF zf	CTGCTGATAATCTGGGCAAG
**42**	vcam1aR zf	TCACCAAGCTTTACTGAGGC
**43**	cdh5 zf F	GGA CAG AGA GCA AGA ATC CT
**44**	cdh5 zf R	CTCACAGACATACACGTAGC
**45**	claudin 11a zf F	ATCACTGCATCACACTCACG
**46**	claudin 11a zf R	GATCGCTTGTTCTTGGCACT
**47**	*claudin 5a zf F*	CCA GAT GCA ATG CAA AGT GC
**48**	*claudin 5a zf R*	AGTGGCACAAGCACGAAGAT
**49**	iNOS_zfF nos2a	CTGAACTGATCTTGGAGGTC
**50**	iNOS_zfRa	ACCACCAACTTCCATGAGCA
**51**	iNOS_zfF nos2b	GGAACTTCTGTGATACCCAG
**52**	iNOS_zfRb	TGTGCTGCATGAAGGACTCT

**53**	*Claudin-2F	CATGAAGGGTCTTTGGATGG
**54**	*Claudin-2R	ACTGAAGATCCGTCCATGCA
**55**	E-selectinF	GCAGCTTCAGTTGTGCAGAA
**56**	E-selectinR	CATTACTGTCAAGCTGGTGG
**57**	ICAM-1 F	GAGACGAAGGACCTCAACAT
**58**	ICAM-1 R	GGTTTGCCTTCACTGTACAG
**59**	T7+sgRNA scar-6	TAATACGACTCACTATAGAAATACGGCGTCTACACACGTTTTAG AGCTAGAAATAGC
**60**	scar-6 CR F	CCAGGAGGAGAAAGATGCAT
**61**	scar-6 CR R	TTATTGGTGTCTTGTGCCGC
**62**	BS-scar-6 F	TGGTGATATATTAAATATATTTTTTATGTT
**63**	BS-scar-6 R	CTTTCCTCAAAAAAATCAAACTCTT
**64**	prdm14 chip F_scar-6	AGGTGTGGTGAGCTGGGG
**65**	prdm14 chip R_scar-6	GTCTCTGGCATGACTTTCGT
**66**	prozb full length R	TCAGGGTTGCTCCCTCTCGG
**67**	prozb full length F +t7	TAATACGACTCACTATAGGATGGAGTCGCTTGTATATCG
**68**	scar-6 morpholino	GCATTTAATTACTCAGTCCACTAACAGC

Appendix Figures and Legends

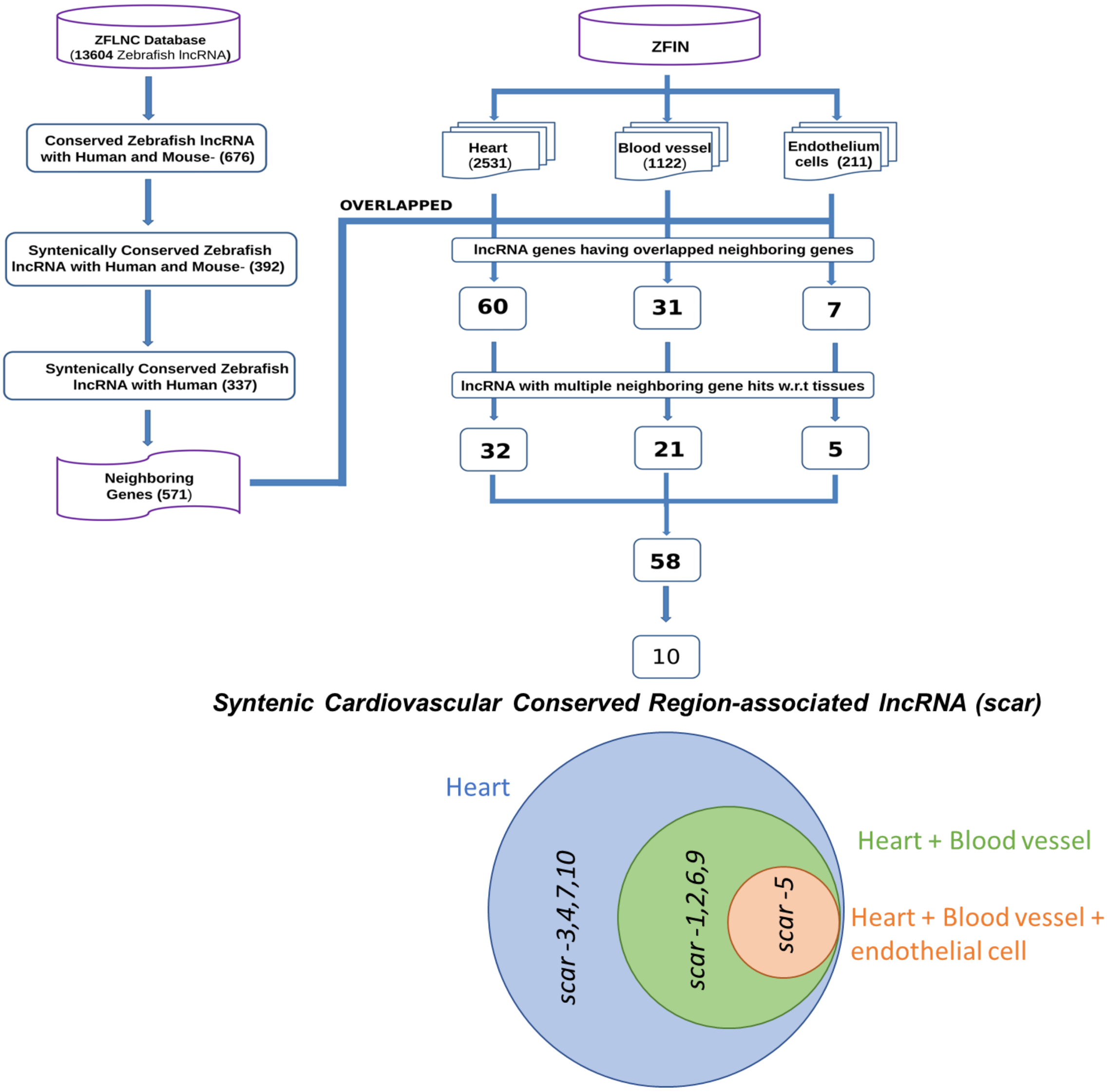

Appendix Figure S1: - Schematic of pipeline used for subset selection of syntenic lncRNAs with its neighbouring protein-coding genes associated in the cardiovascular system.

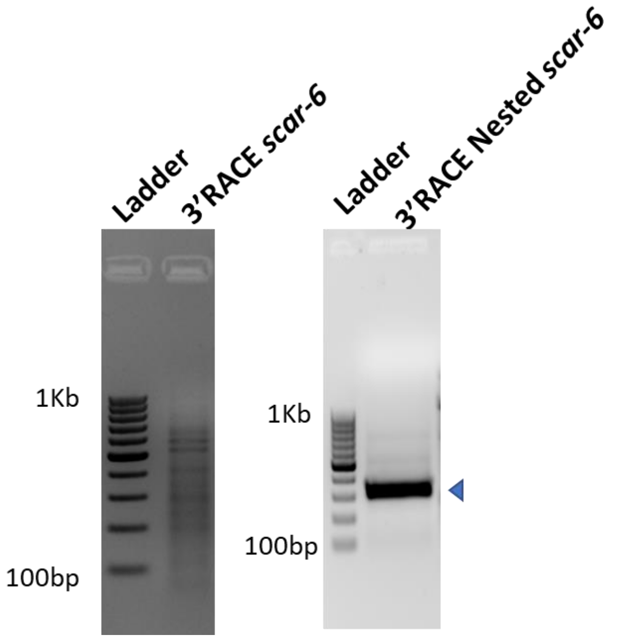

Appendix Figure S2: - Agarose gel image for 3'-RACE of *scar-6* lncRNA followed by nested PCR resulted in expected size of the transcript.

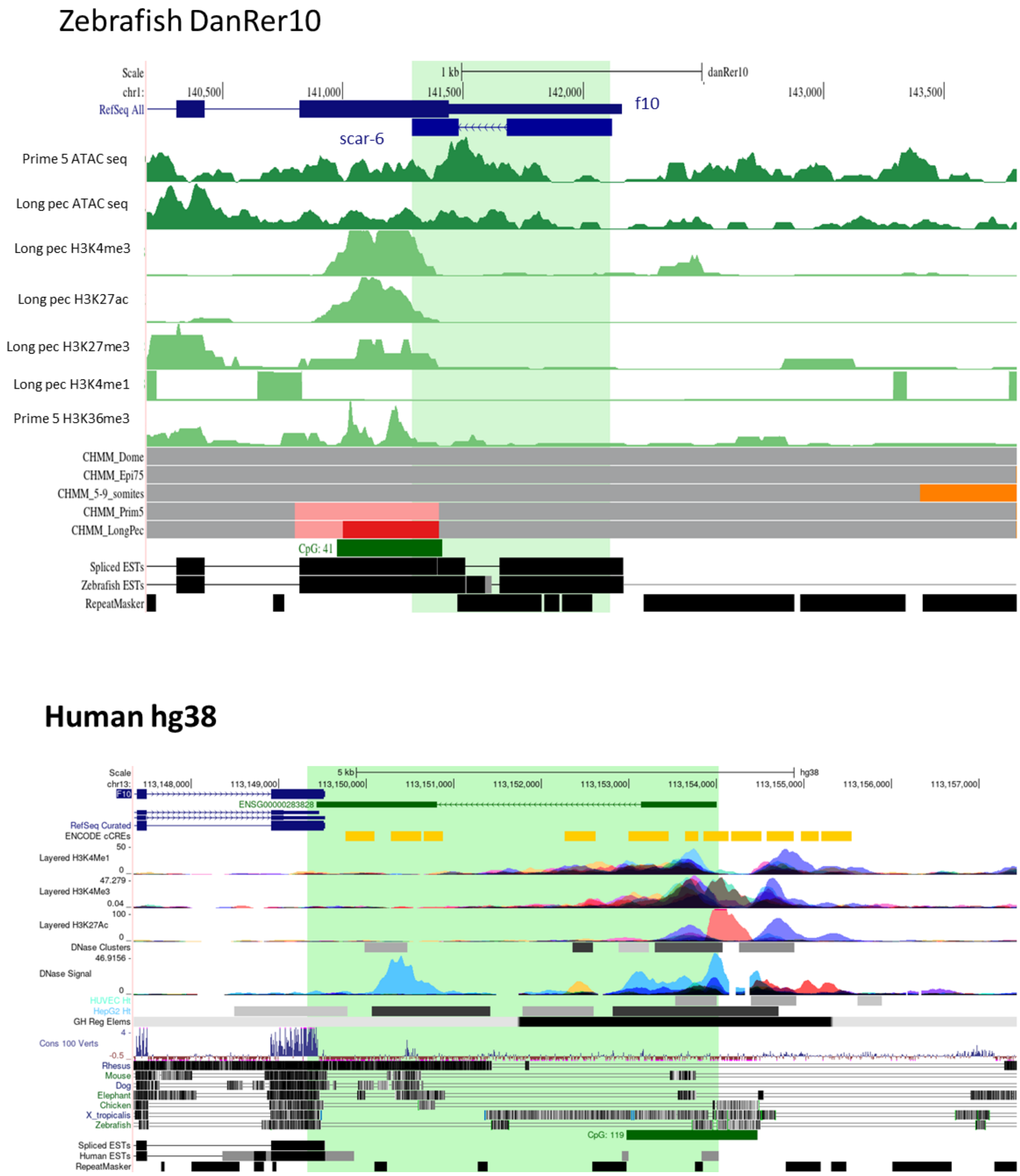

Appendix Figure S3: - Genomic context of *scar-6* locus in zebrafish and Human with H3K4me1, H3K4me3, H3K27ac, H3K36me3, H3K27me3, chromatin state marks in UCSC genome browser (ENCODE Project Consortium *et al*, 2020; Baranasic *et al*, 2022)

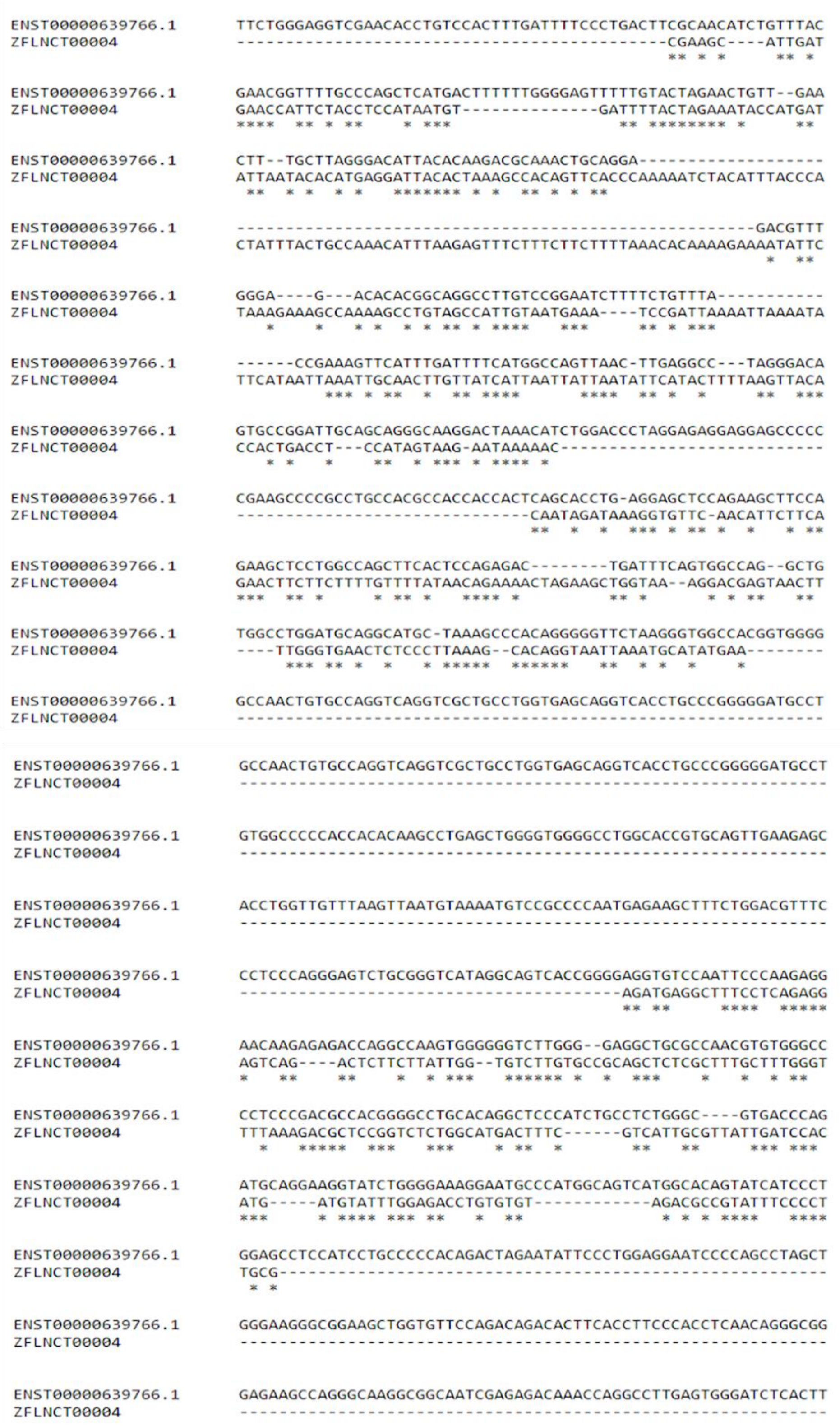

Appendix Figure S4: - ClusterW based sequence alignment of zebrafish *scar-6 (ZFLNCT00004)* and human *SCAR-6 (ENST00000639766.1)* lncRNA transcript.

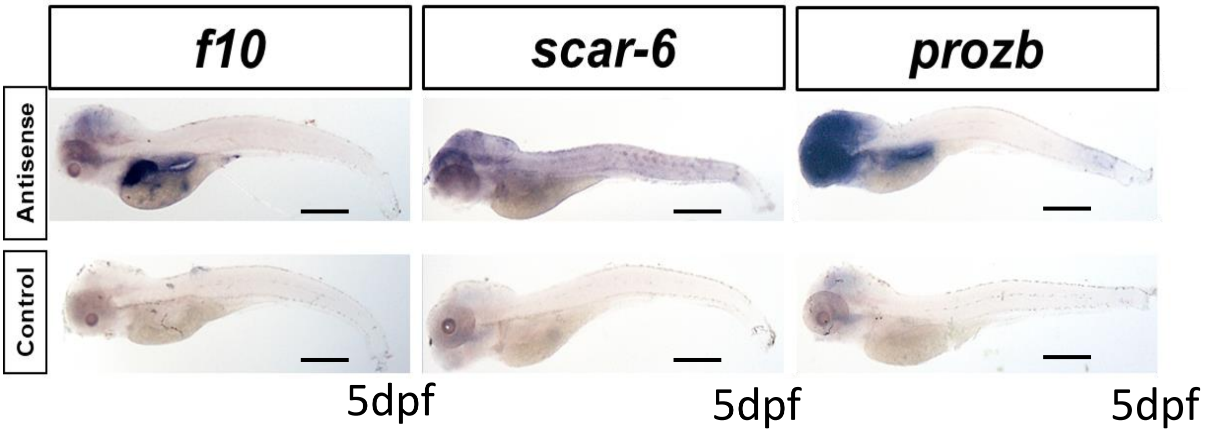

Appendix Figure S5: -Whole mount in-situ hybridization expression analysis of *scar-6, f10,* and *prozb* transcripts of zebrafish in 5dpf embryo. Sense probes were used as controls for *f10* and *prozb.* No probe control was used for *scar-6.* 4 x magnification; scale bar = 500 µm

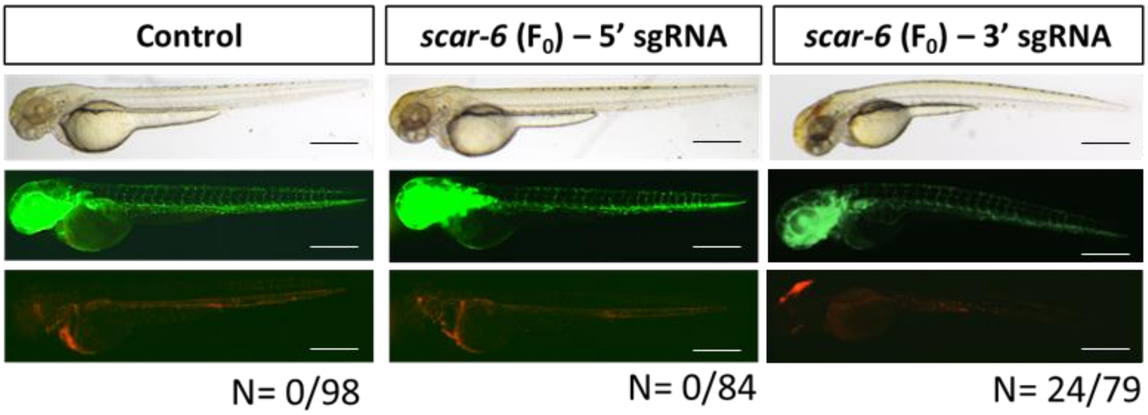

Appendix Figure S6: - Representative image showing transgenic gib004Tg(*fli1a*:EGFP;*gata1a*:DsRed) 3dpf zebrafish injected with RNP complex of CRISPR-Cas9 targeting 5' and 3' region of *scar-6* gene. 2.5x magnification, scale bar = 500 µm

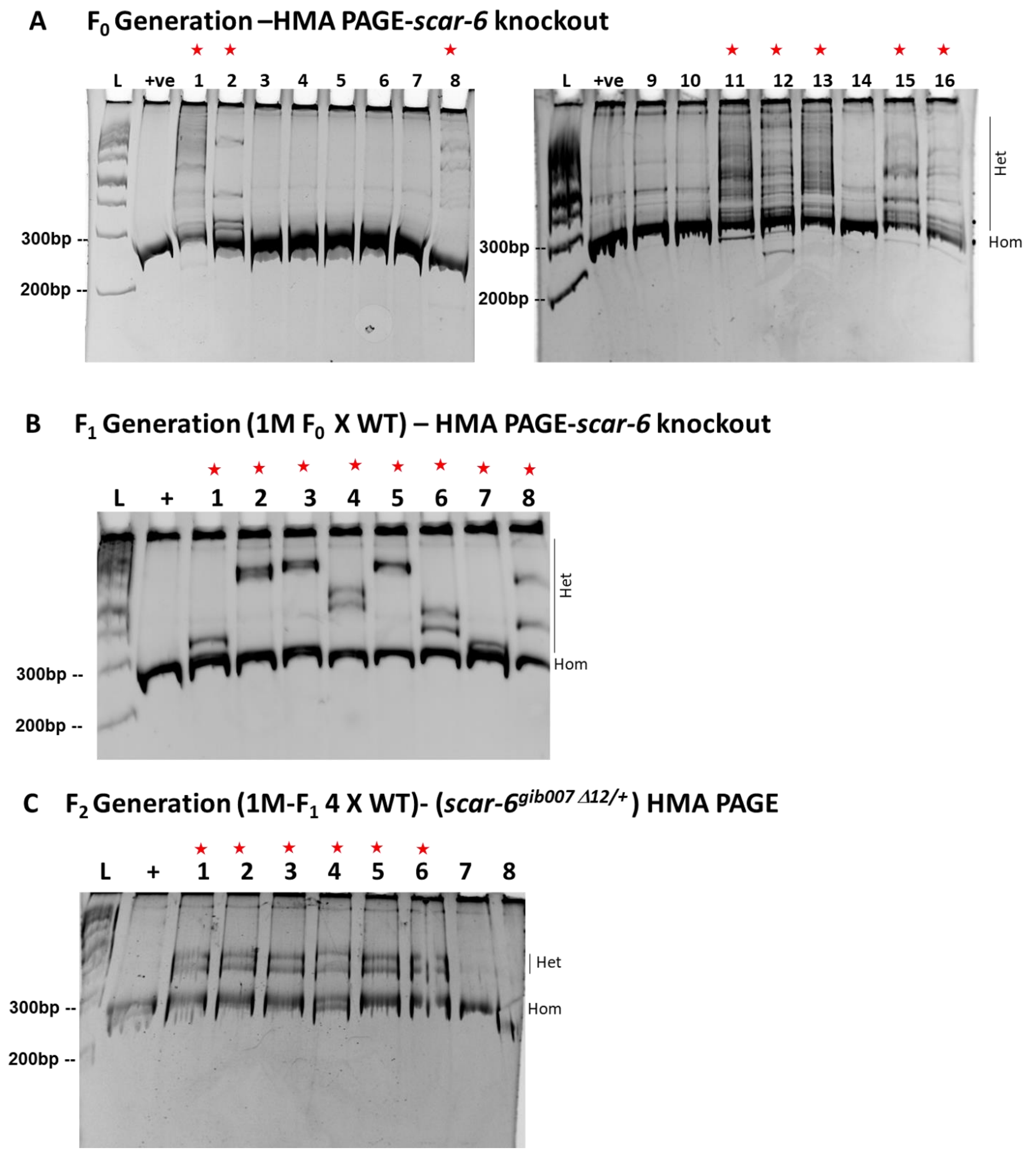

Appendix Figure S7: - CRISPR-Cas9 mediated mutant of *scar-6* lncRNA

[A] Heteroduplex mobility assay (HMA) PAGE gel for *scar-6* target region in F_0 zebrafish.

[B] HMA-PAGE gel for *scar-6* target region in F_1 zebrafish. Raw image of Figure EV2A (star represents positive for mutation)

[D] HMA-PAGE gel for *scar-6* target region in F_2 zebrafish. (star represents positive for mutation).; Het- Heteroduplex, Hom- Homoduplex.

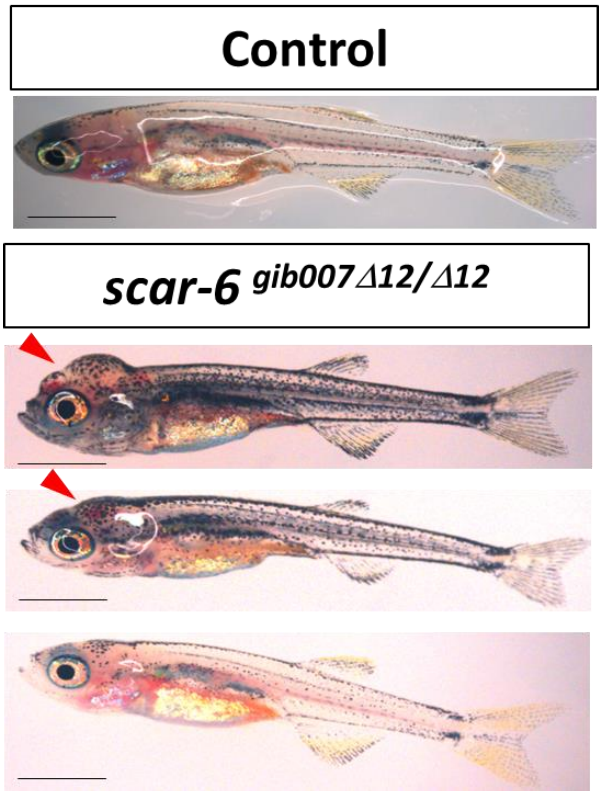

Appendix Figure S8: - Raw image of Figure 3 D-E of 30 dpf zebrafish from wild type and *scar-6*$^{gib007Δ12/Δ12}$ animals, which were closely monitored for their survival. 1.5x magnification; scale bar - 1mm

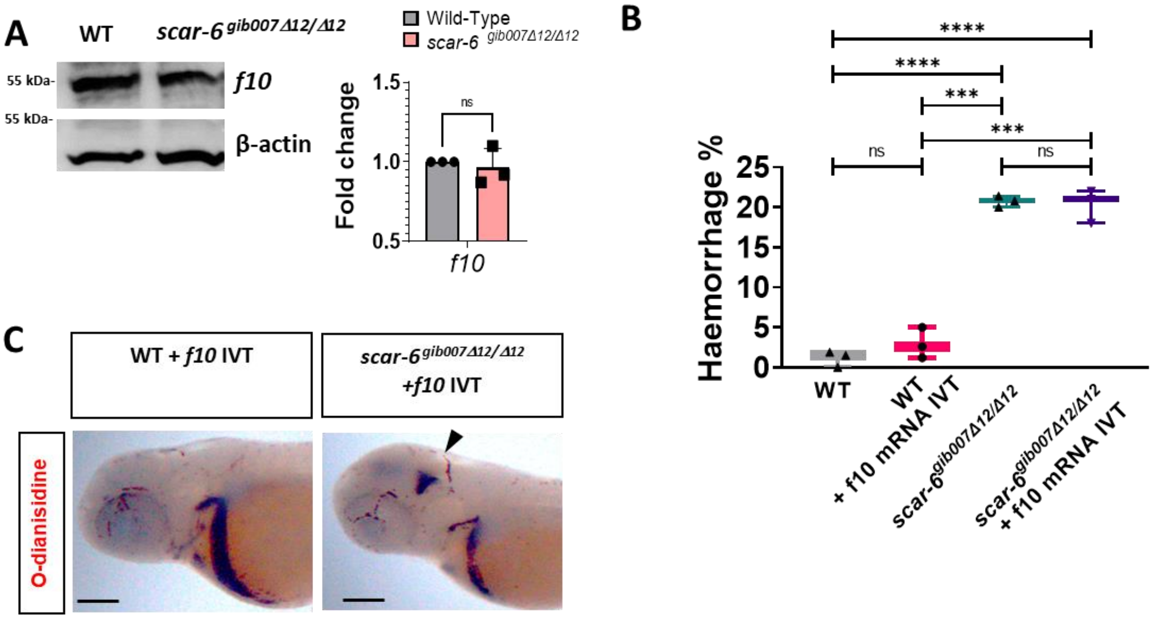

Appendix Figure S9: Rescue of *scar-6* mutant with *f10* mRNA

[A]Western blot of *f10* in wild type and *scar-6*$^{gib007\Delta12/\Delta12}$ mutant zebrafish. The bar plot represents the quantification of the western blot from 3 independent biological replicates plotted as mean fold change ± standard deviation. ns- not significant (two-tailed unpaired t-test)

[B]Box plot representing the percentage of animals exhibiting haemorrhage phenotype in wild type(WT), WT injected with f10 IVT RNA (100 ng/uL), *scar-6*$^{gib007\Delta12/\Delta12}$ mutant and *scar-6*$^{gib007\Delta12/\Delta12}$ mutant zebrafish injected with f10 IVT RNA (100 ng/uL). Data from 3 independent biological replicates plotted as mean percentage ± standard deviation; ns- not significant; *** p<0.001, **** p<0.0001 (two-tailed unpaired t-test).

[C]Representative image showing the cranial region of 3dpf zebrafish with o-dianisidine staining of RBC blood cells in wild type and *scar-6*$^{gib007\Delta12/\Delta12}$ mutant zebrafish injected with f10 IVT RNA (100 ng/uL). Black arrowhead denotes haemorrhage. 4x magnification; scale bar - 200 μm

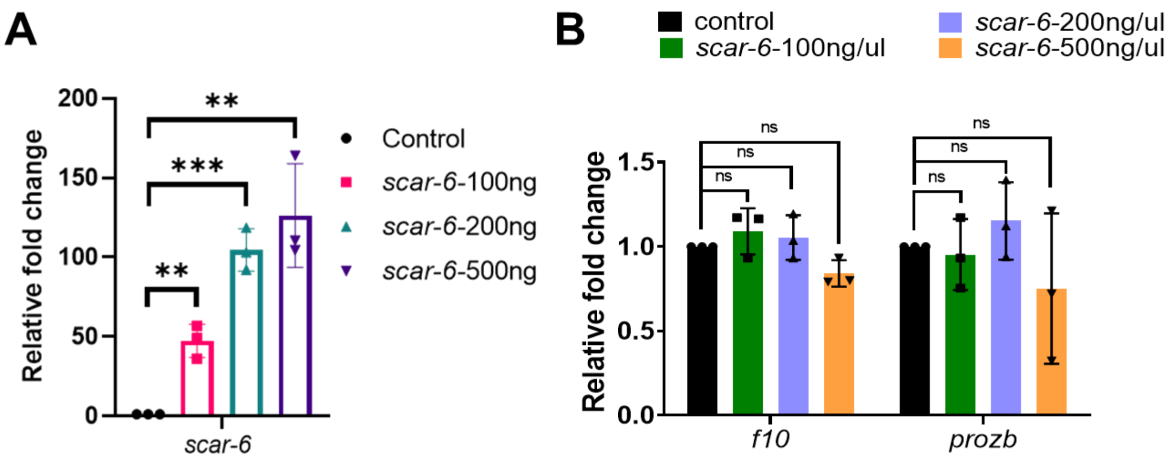

Appendix Figure S10: -Overexpression of *scar-6* lncRNA in zebrafish

[A] Relative fold change expression of *scar-6* at 3dpf upon *scar-6* IVT RNA overexpression in zebrafish at different concentrations (100, 200 500 ng/uL). Data from 3 independent biological replicates plotted as mean fold change ± standard deviation. ** P<0.01, *** p<0.001, **** p<0.0001 (two-tailed unpaired t-test).

[B] Relative fold change expression of *f10*, and *prozb* upon *scar-6* IVT RNA overexpression in zebrafish at different concentrations (100, 200 500 ng/uL). Data from 3 independent biological replicates plotted as mean fold change ± standard deviation. ns - not significant (two-tailed unpaired t-test).

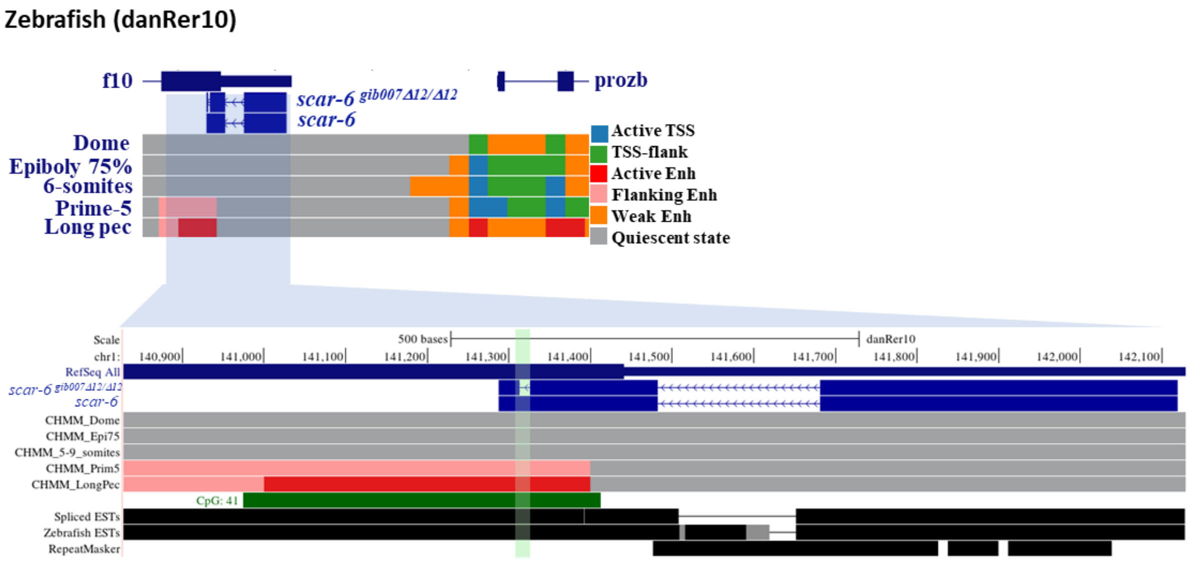

Appendix Figure S11:- UCSC genome browser snapshot of zebrafish *scar-6* locus with chromatin annotation marks from danio-code data across 5 developmental stages.

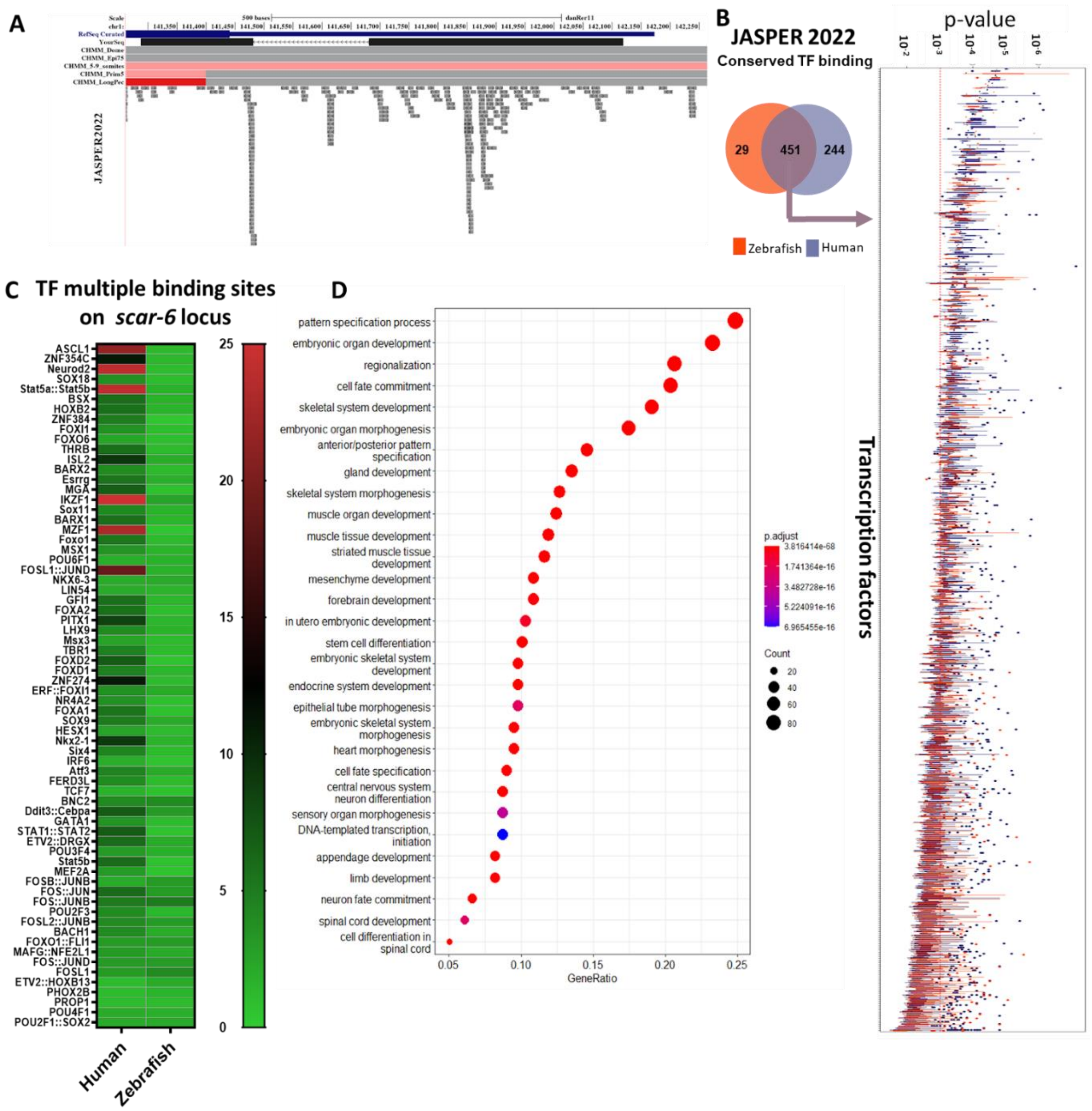

Appendix Figure S12: - Cis-Regulatory feature of *scar-6* locus

[A]UCSC genome browser screenshot of *scar-6* locus and enrichment of transcription factors (TF) from JASPER 2022 data set with p value <0.001.

[B] Overlap of TF from *SCAR-6* locus of human and *scar-6* locus of zebrafish extracted from JASPER 2022 database. Box plot representing TF and their motif p-value. The red line indicates p value = 0.001.l

[C]Heatmap representing the top 64 TF which showed multiple binding sites on *SCAR-6* locus of human and *scar-6* locus of zebrafish.

[D]Biological Gene ontology for common TF between human and zebrafish *scar-6* lncRNA locus.

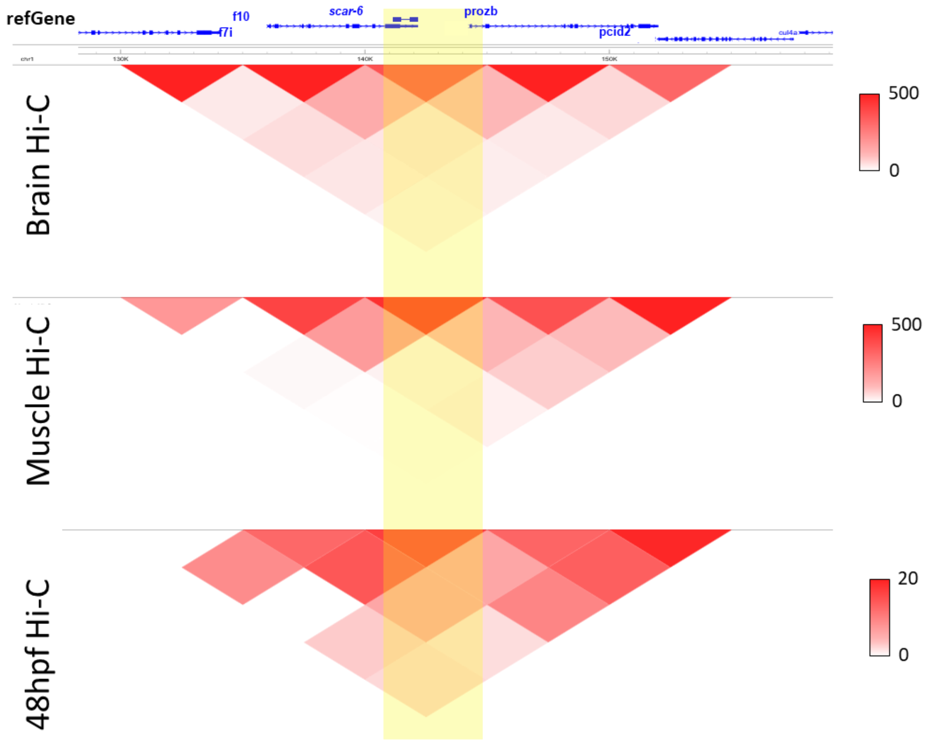

Appendix Figure S13:- Hi-C heatmap representation of zebrafish *scar-6* locus in brain, muscle and 48 hpf zebrafish at 5kb resolution (Yang *et al*, 2020, Franke, M *et al*. 2020).

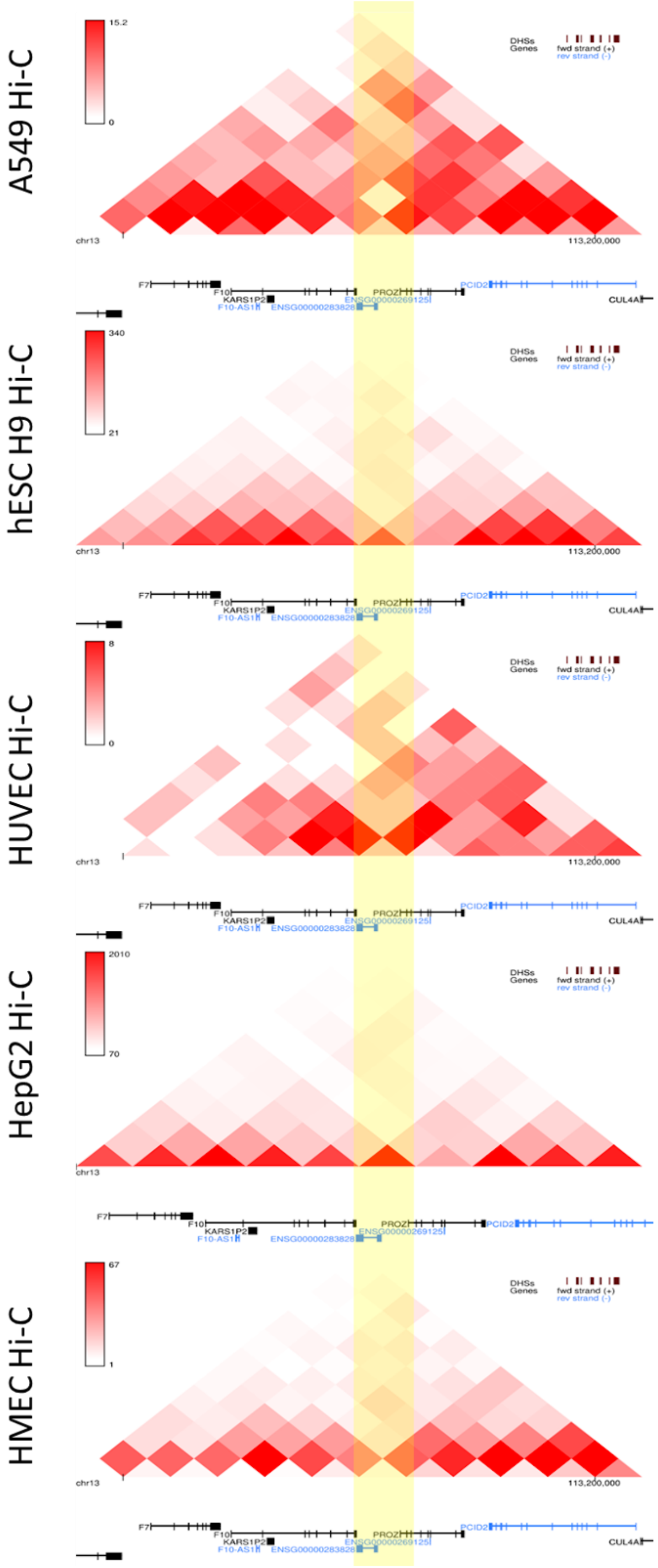

Appendix Figure S14: - Hi-C heatmap representation of Human *SCAR-6* locus in A549, hESC-H9, HUVEC, HepG2 and HMEC at 10kb resolution from ENCODE database (ENCODE Project Consortium *et al*, 2020; Wang *et al*, 2018)

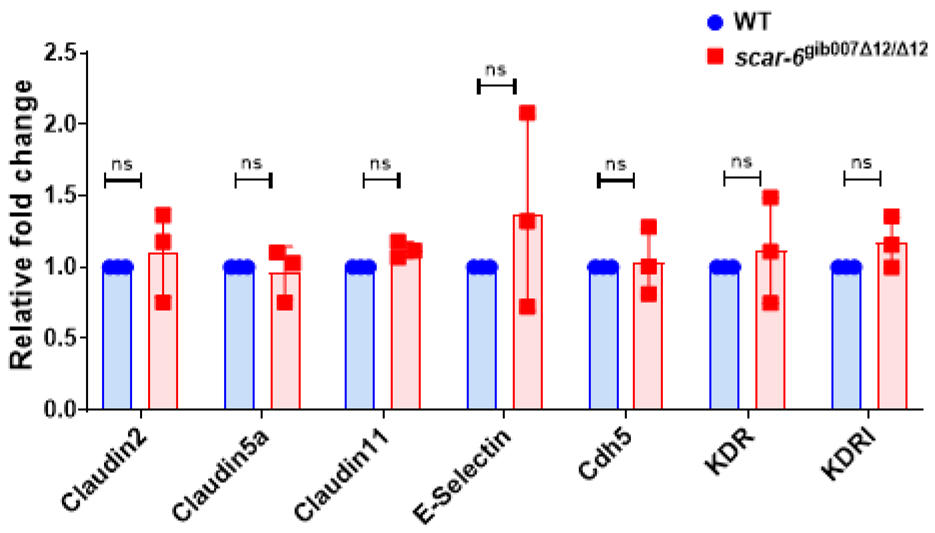

Appendix Figure S15: - qRT-PCR of the endothelial associated gene downstream of Nf-kB. when compared between wild type and *scar-6*$^{gib007\Delta12/\Delta12}$ mutant zebrafish. Data from 3 independent biological replicates plotted as mean fold change ± standard deviation; ns- not significant (two-tailed unpaired t-test).

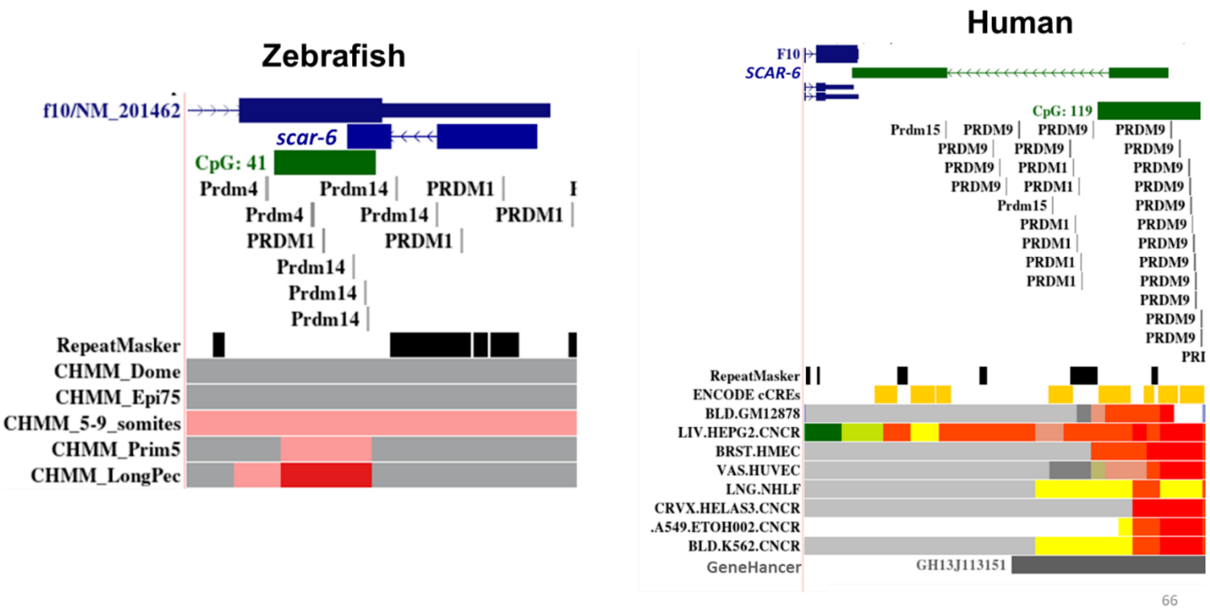

Appendix Figure S16 : - UCSC genome browser screenshot of zebrafish *scar-6* and human *SCAR-6* locus depicting TF binding motifs of *PRDM* family proteins from JASPER 2022 database.

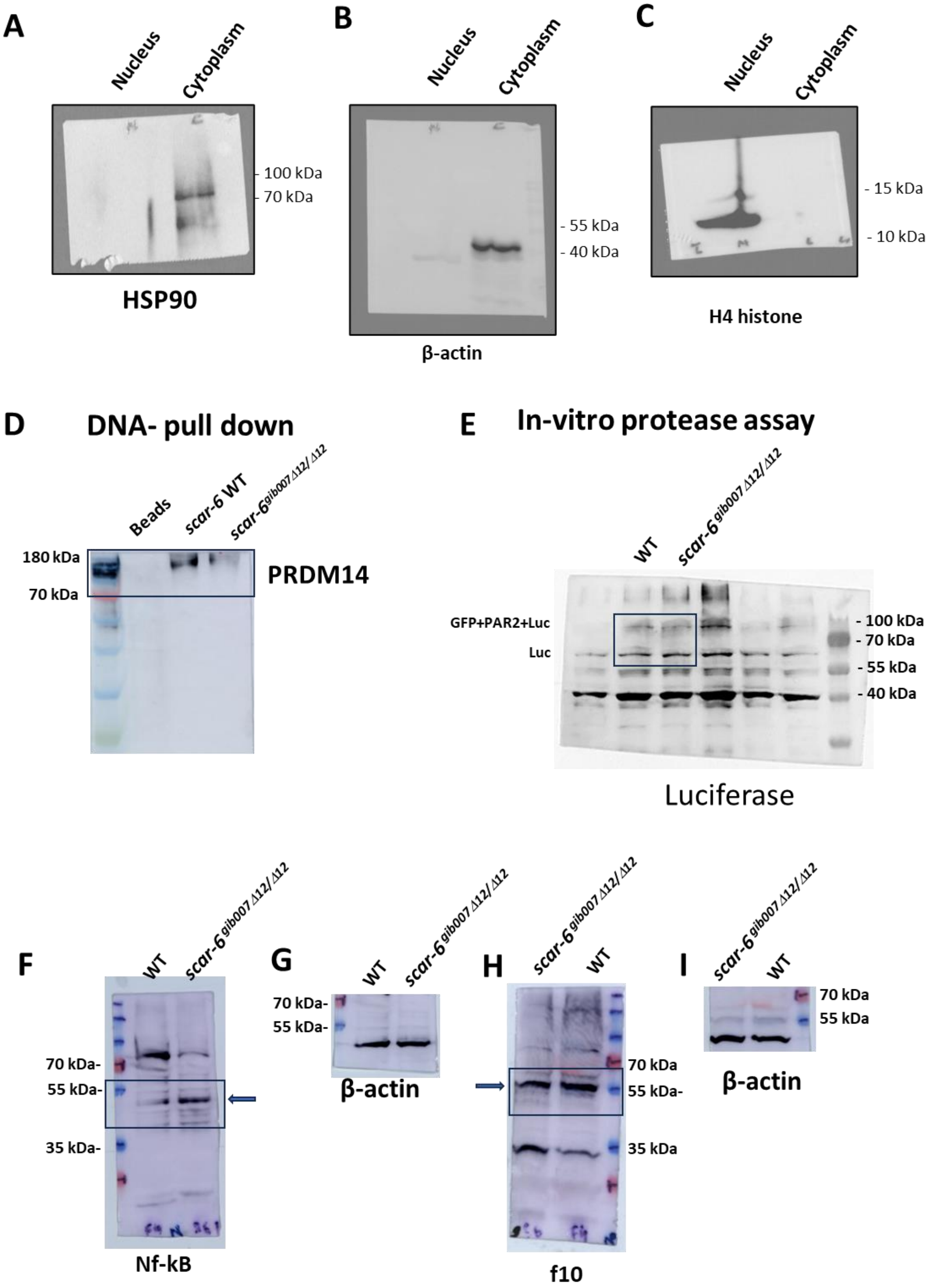

Appendix Figure S17 : -Raw images for western blot

[A, B, C] Sub-cellular fractionation assay probing for HSP90, β-actin and H4 histone antibody respectively

[D] DNA pulldown experiment of *scar-6* locus in wild type and *scar-6*$^{gib007\Delta12/\Delta12}$ mutant DNA probing with *PRDM14* antibody.

[E] In-vitro protease assay probing with luciferase antibody.

[F-G] Western blot in wildtype and *scar-6*$^{gib007\Delta12/\Delta12}$ mutant zebrafish for Nf-κB and β-actin.

[H-I] Western blot in wildtype and *scar-6*$^{gib007\Delta12/\Delta12}$ mutant zebrafish for *f10* and β-actin.
