# Supplementary figures and images for: SCAR-6 elncRNA locus epigenetically regulates PROZ and modulates coagulation and vascular function
Source: EMBO Rep. 2024 Oct 2;25(11):4950–78. doi: 10.1038/s44319-024-00272-w (PMC11549340; doi:10.1038/s44319-024-00272-w)

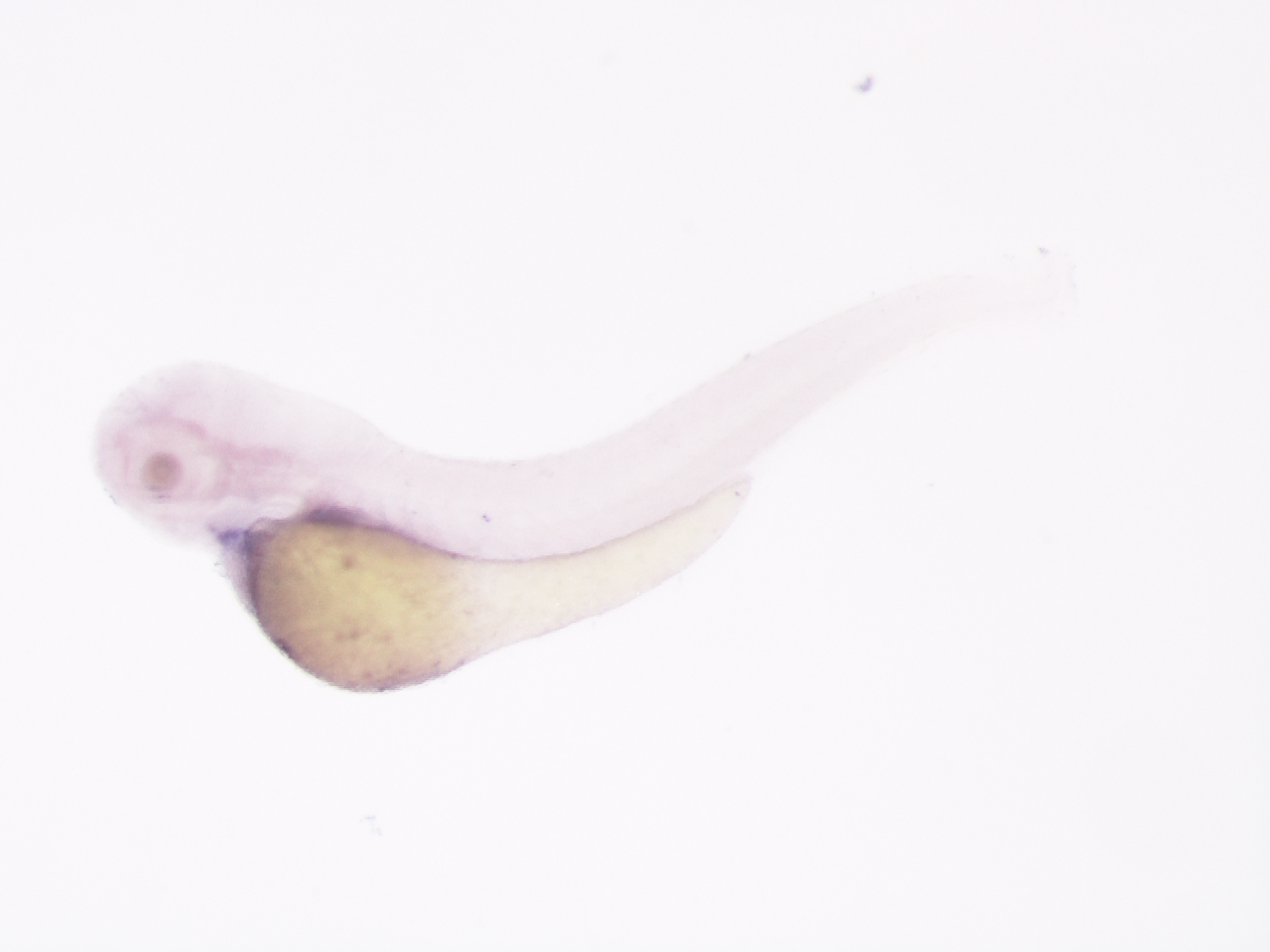

Supplement: Supplementary file 3 — Source data Fig. 1 [file 44319_2024_272_MOESM3_ESM.zip › Figure 1/1F/f10_AS.tif]

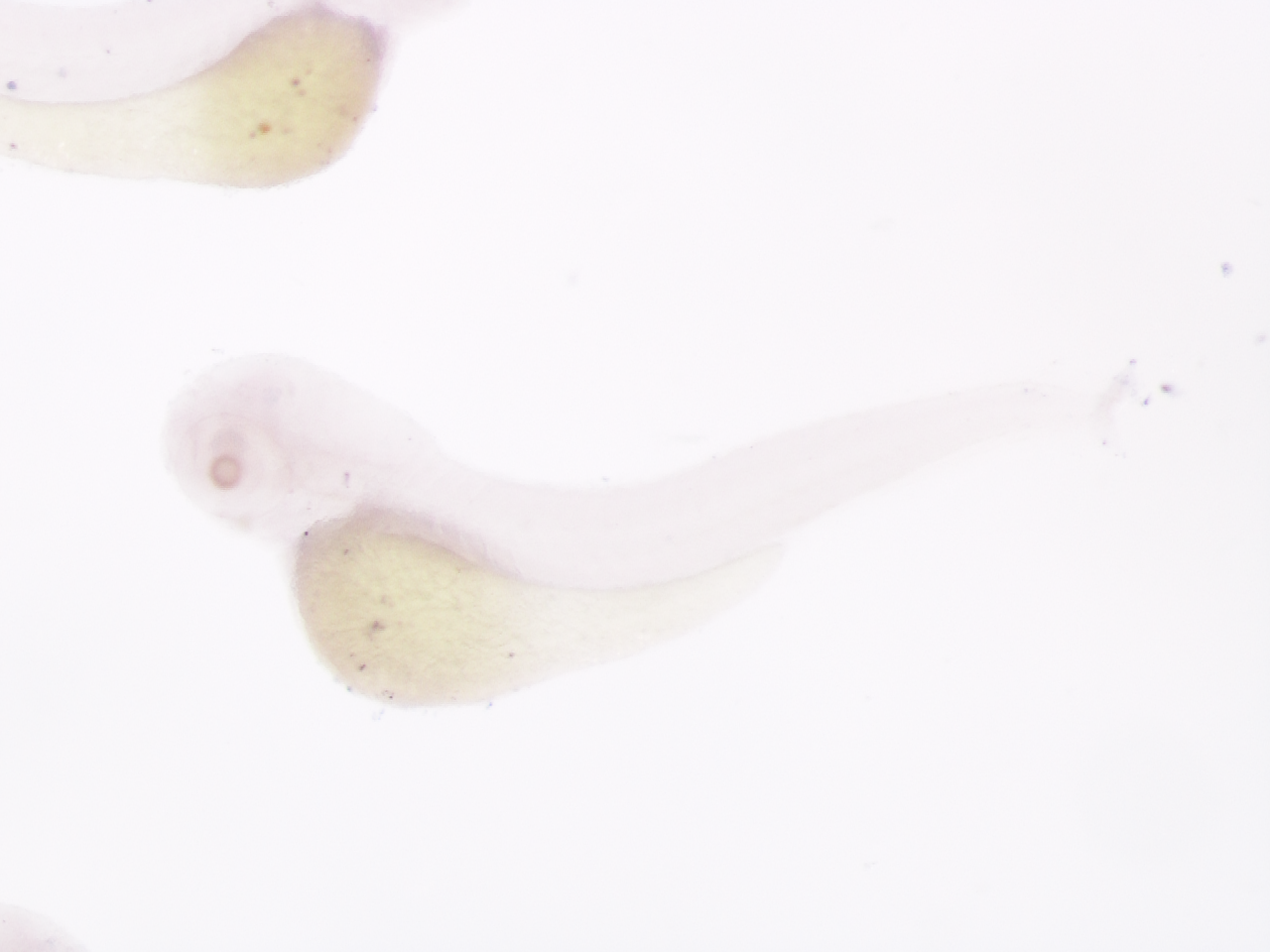

Supplement: Supplementary file 3 — Source data Fig. 1 [file 44319_2024_272_MOESM3_ESM.zip › Figure 1/1F/f10_s.tif]

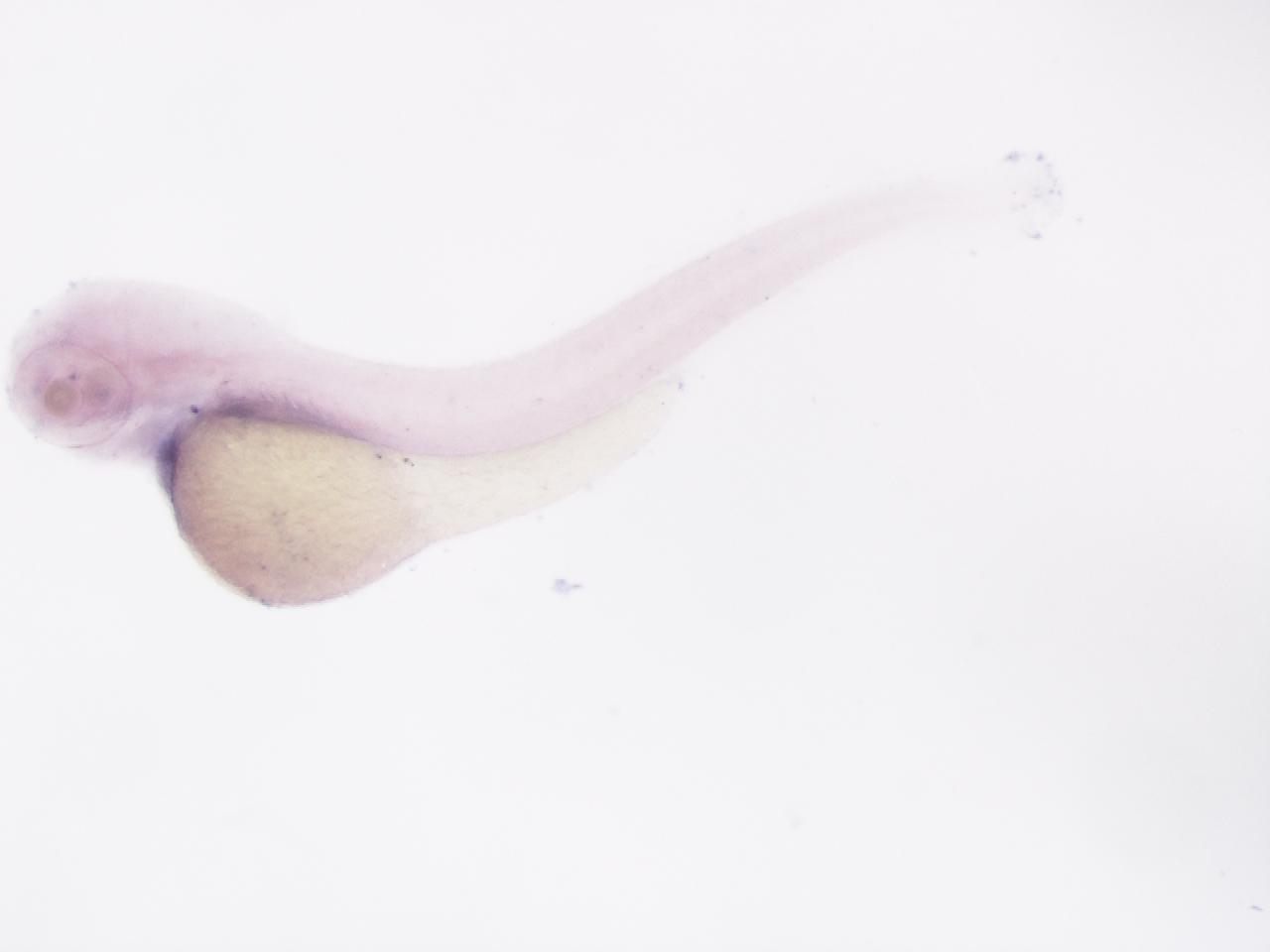

Supplement: Supplementary file 3 — Source data Fig. 1 [file 44319_2024_272_MOESM3_ESM.zip › Figure 1/1F/prozb_AS.tif]

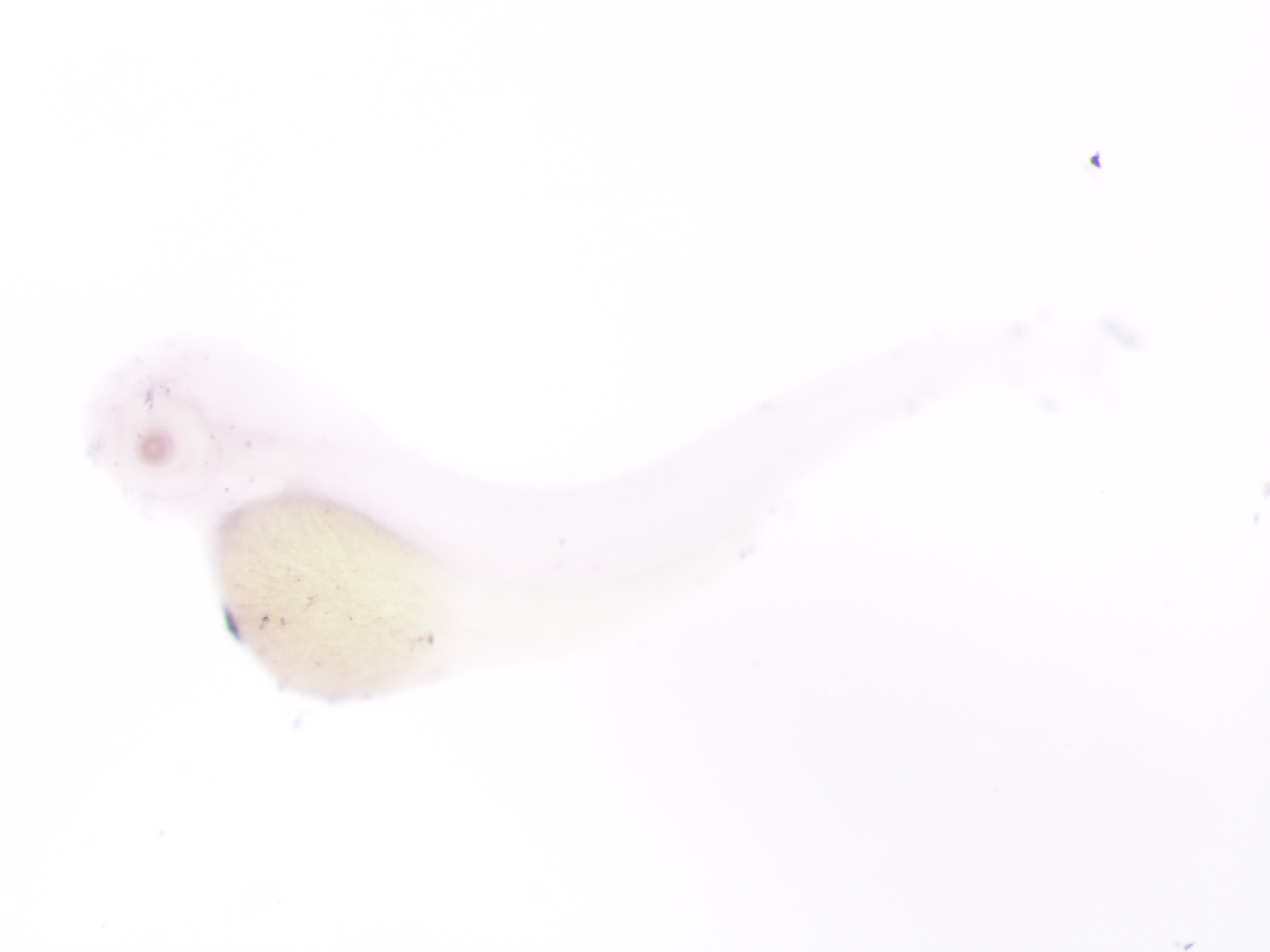

Supplement: Supplementary file 3 — Source data Fig. 1 [file 44319_2024_272_MOESM3_ESM.zip › Figure 1/1F/prozb_s.tif]

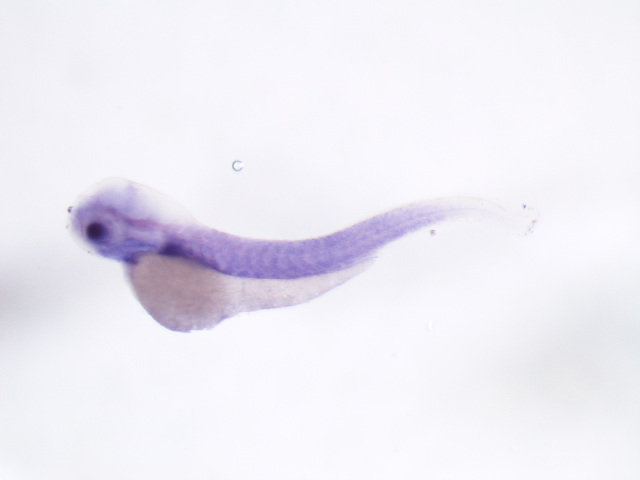

Supplement: Supplementary file 3 — Source data Fig. 1 [file 44319_2024_272_MOESM3_ESM.zip › Figure 1/1F/scar6_AS.tif]

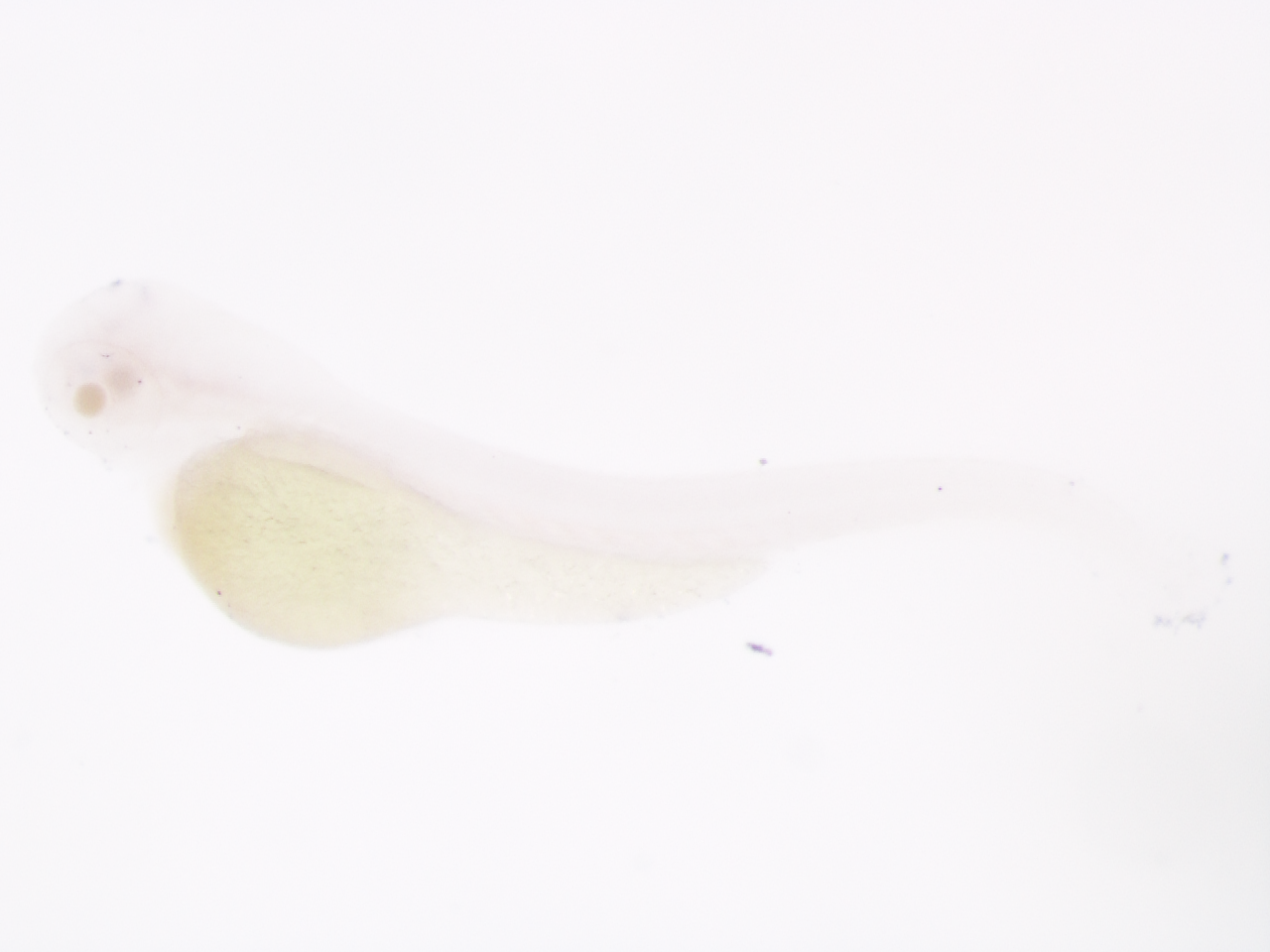

Supplement: Supplementary file 3 — Source data Fig. 1 [file 44319_2024_272_MOESM3_ESM.zip › Figure 1/1F/scar6_s.tif]

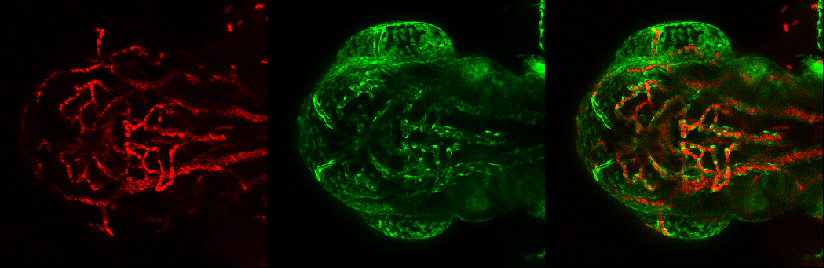

Supplement: Supplementary file 4 — Source data Fig. 2 [file 44319_2024_272_MOESM4_ESM.zip › Figure 2/2F/control.tif]

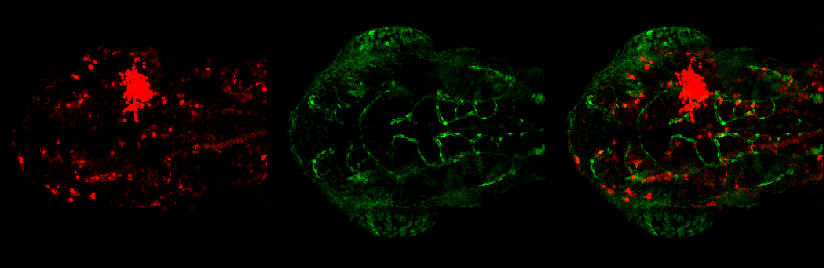

Supplement: Supplementary file 4 — Source data Fig. 2 [file 44319_2024_272_MOESM4_ESM.zip › Figure 2/2F/scar-6 del12 mutant.tif]

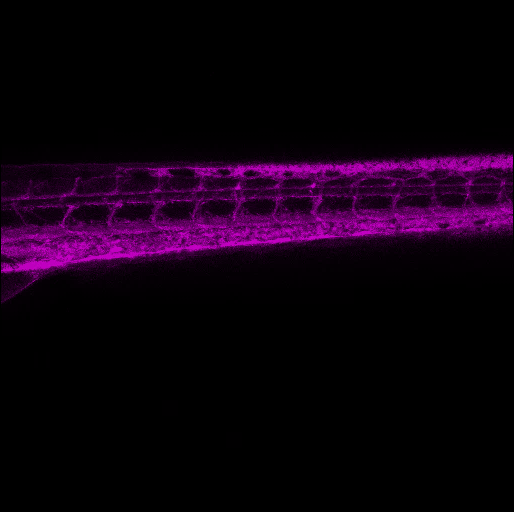

Supplement: Supplementary file 4 — Source data Fig. 2 [file 44319_2024_272_MOESM4_ESM.zip › Figure 2/2H/control.tif]

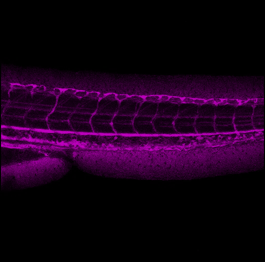

Supplement: Supplementary file 4 — Source data Fig. 2 [file 44319_2024_272_MOESM4_ESM.zip › Figure 2/2H/scar-6del12.tif]

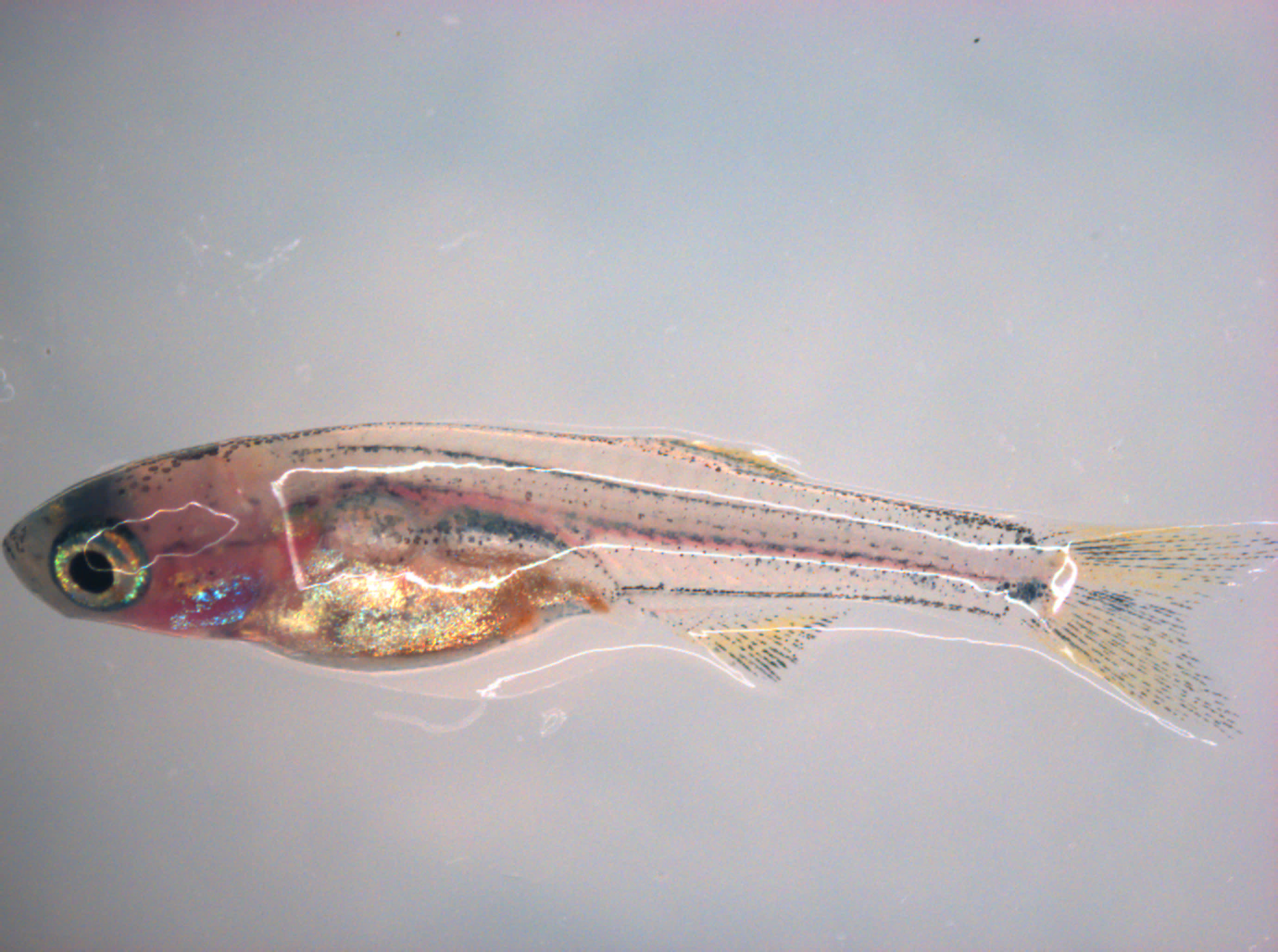

Supplement: Supplementary file 5 — Source data Fig. 3 [file 44319_2024_272_MOESM5_ESM.zip › Figure 3/3D/Control.tif]

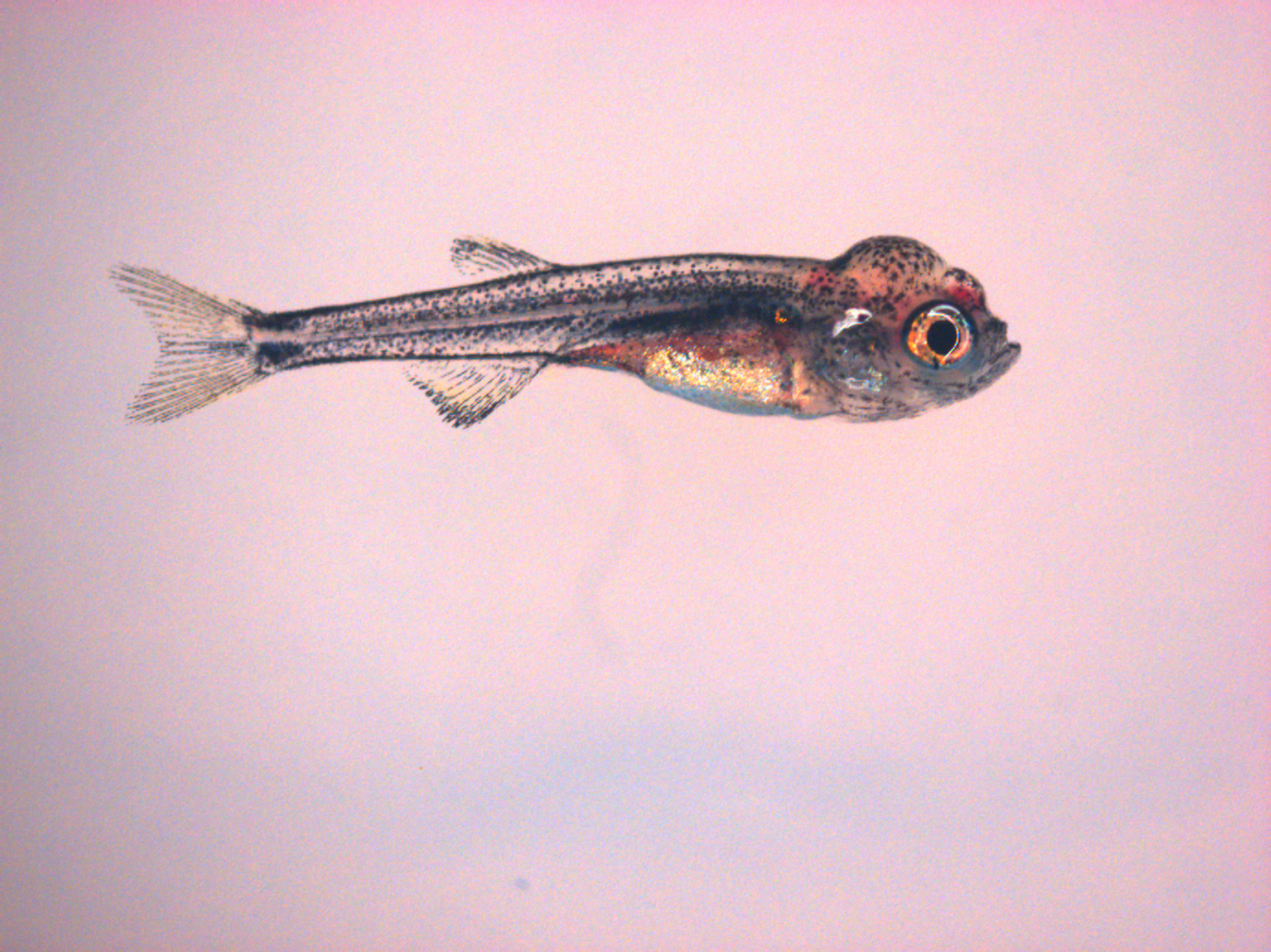

Supplement: Supplementary file 5 — Source data Fig. 3 [file 44319_2024_272_MOESM5_ESM.zip › Figure 3/3E/scar-6 del12 (1).tif]

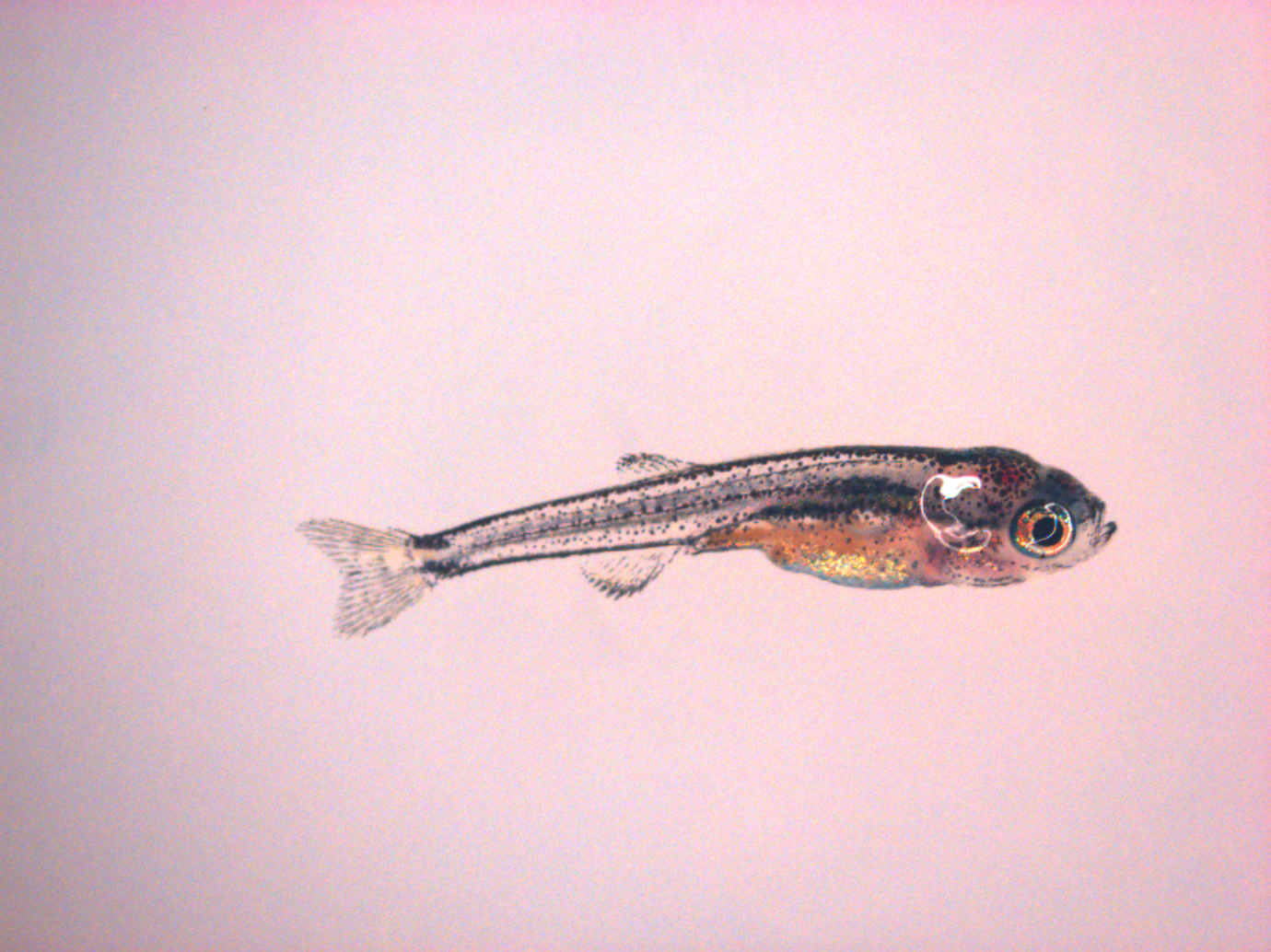

Supplement: Supplementary file 5 — Source data Fig. 3 [file 44319_2024_272_MOESM5_ESM.zip › Figure 3/3E/scar-6 del12 (2).tif]

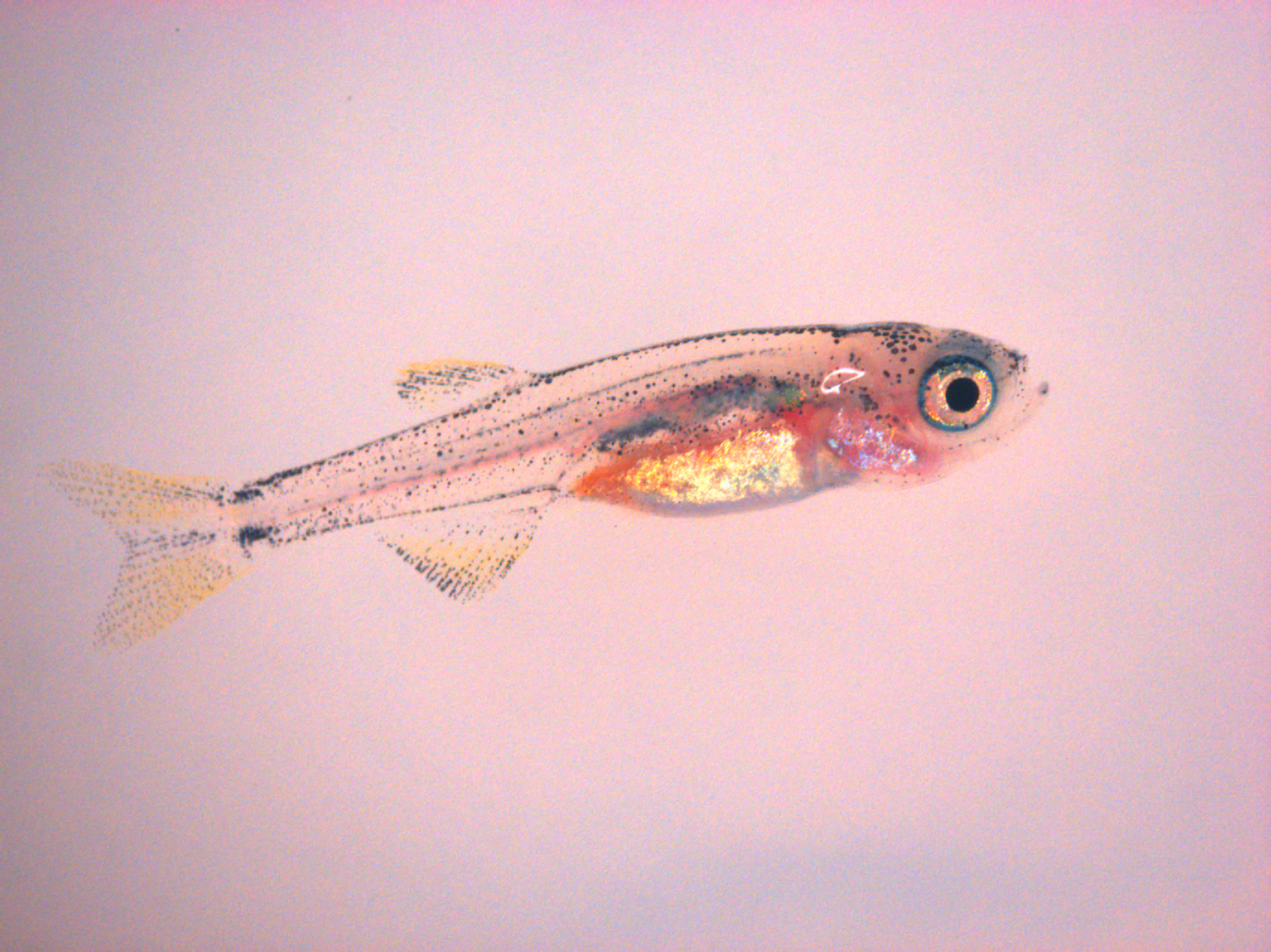

Supplement: Supplementary file 5 — Source data Fig. 3 [file 44319_2024_272_MOESM5_ESM.zip › Figure 3/3E/scar-6 del12 (3).tif]

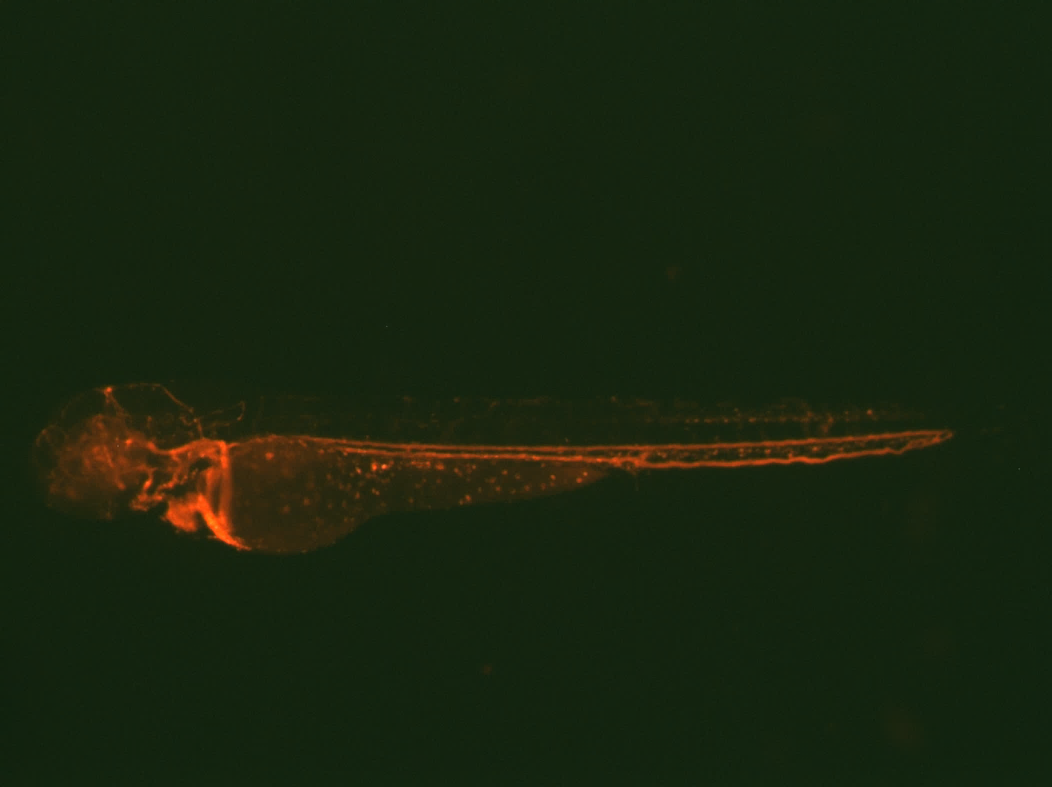

Supplement: Supplementary file 5 — Source data Fig. 3 [file 44319_2024_272_MOESM5_ESM.zip › Figure 3/3G/Control.tif]

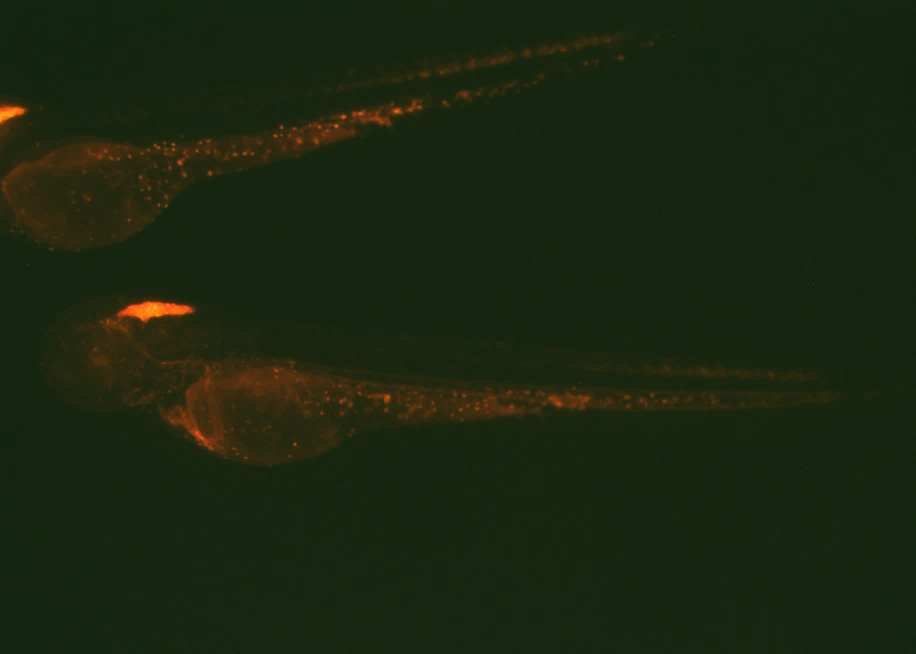

Supplement: Supplementary file 5 — Source data Fig. 3 [file 44319_2024_272_MOESM5_ESM.zip › Figure 3/3G/scar6 del12 rescue.tif]

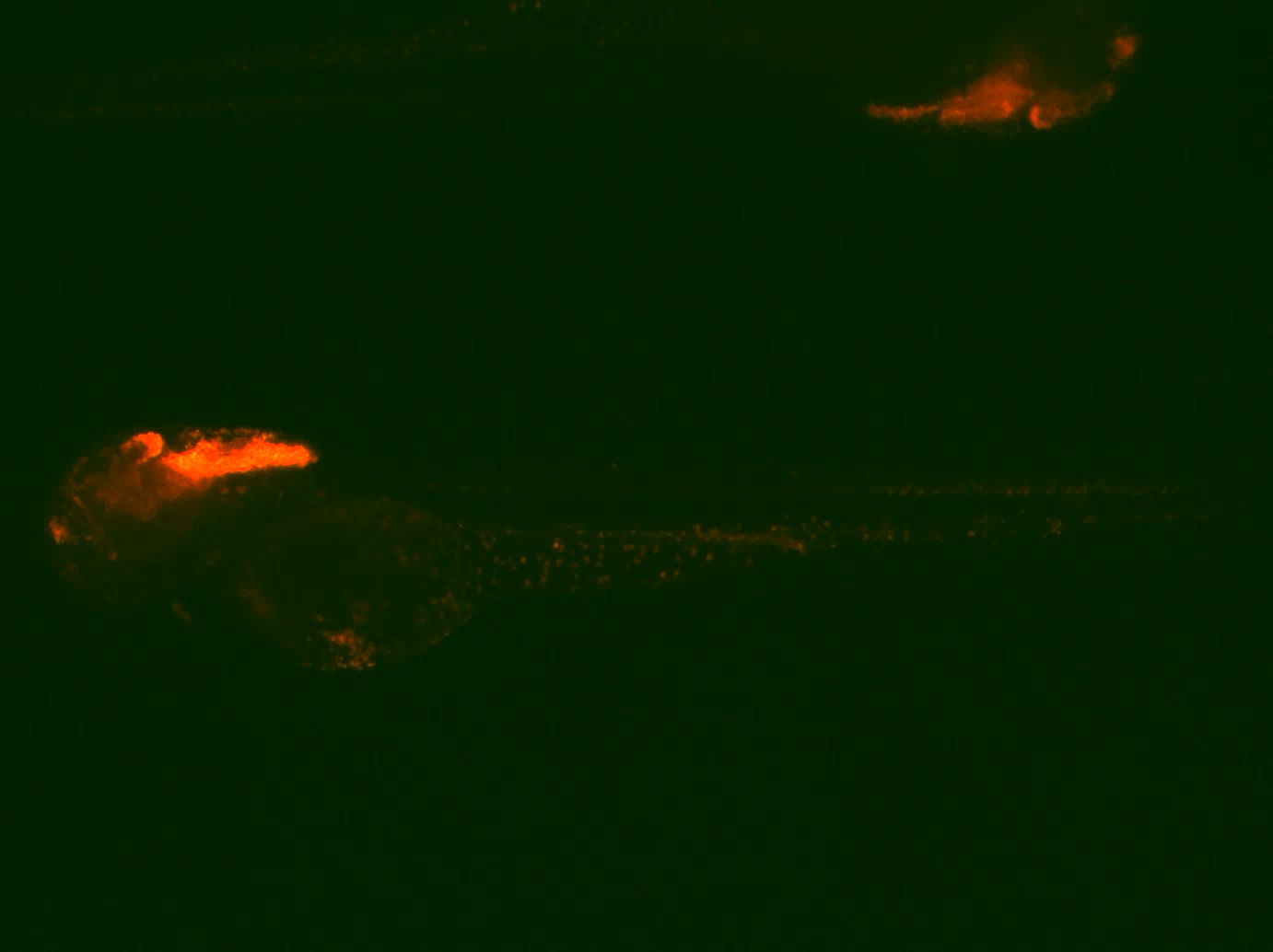

Supplement: Supplementary file 5 — Source data Fig. 3 [file 44319_2024_272_MOESM5_ESM.zip › Figure 3/3G/scar6 del12.tif]

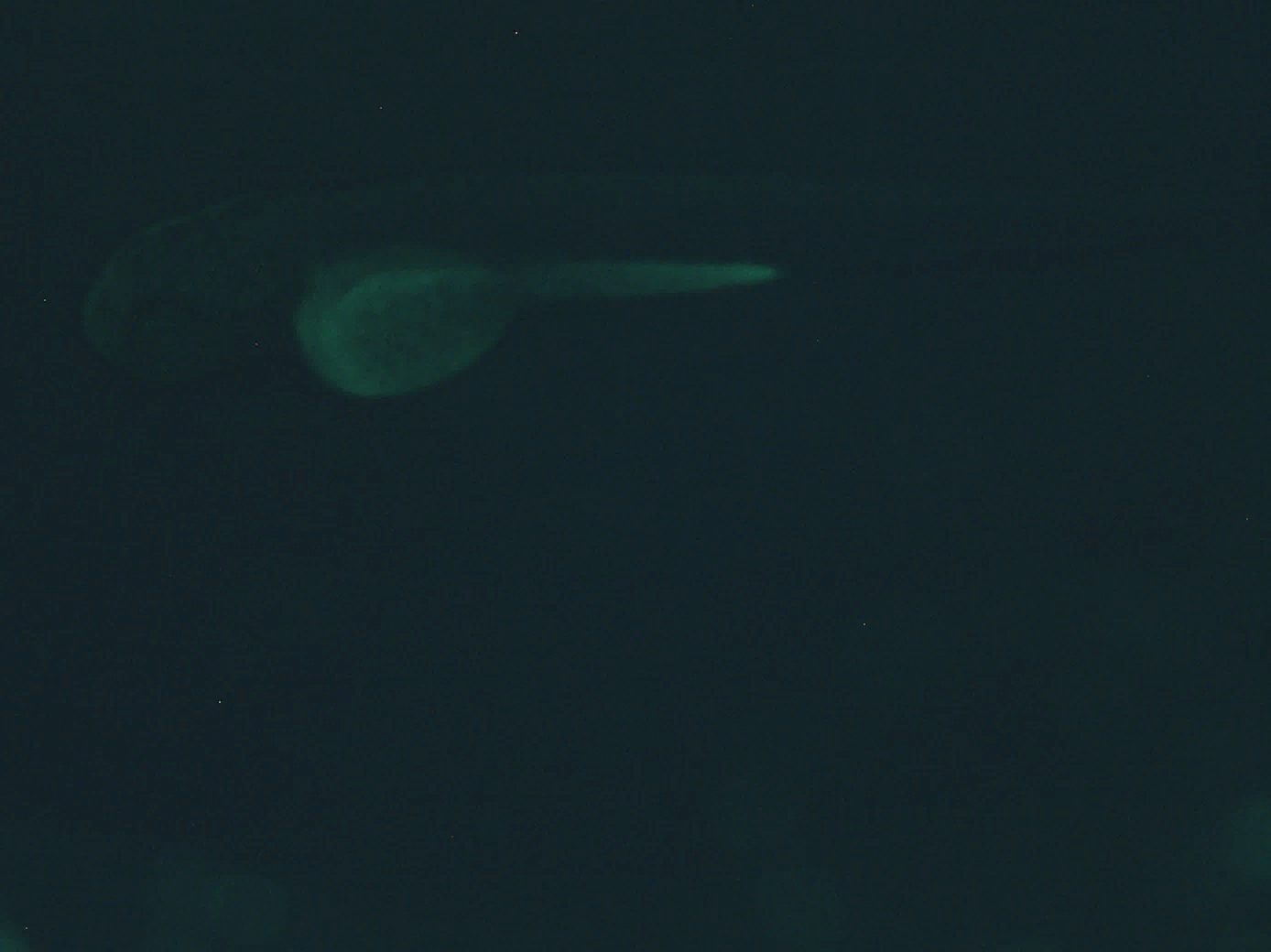

Supplement: Supplementary file 6 — Source data Fig. 4 [file 44319_2024_272_MOESM6_ESM.zip › Figure 4/4C/Control.tif]

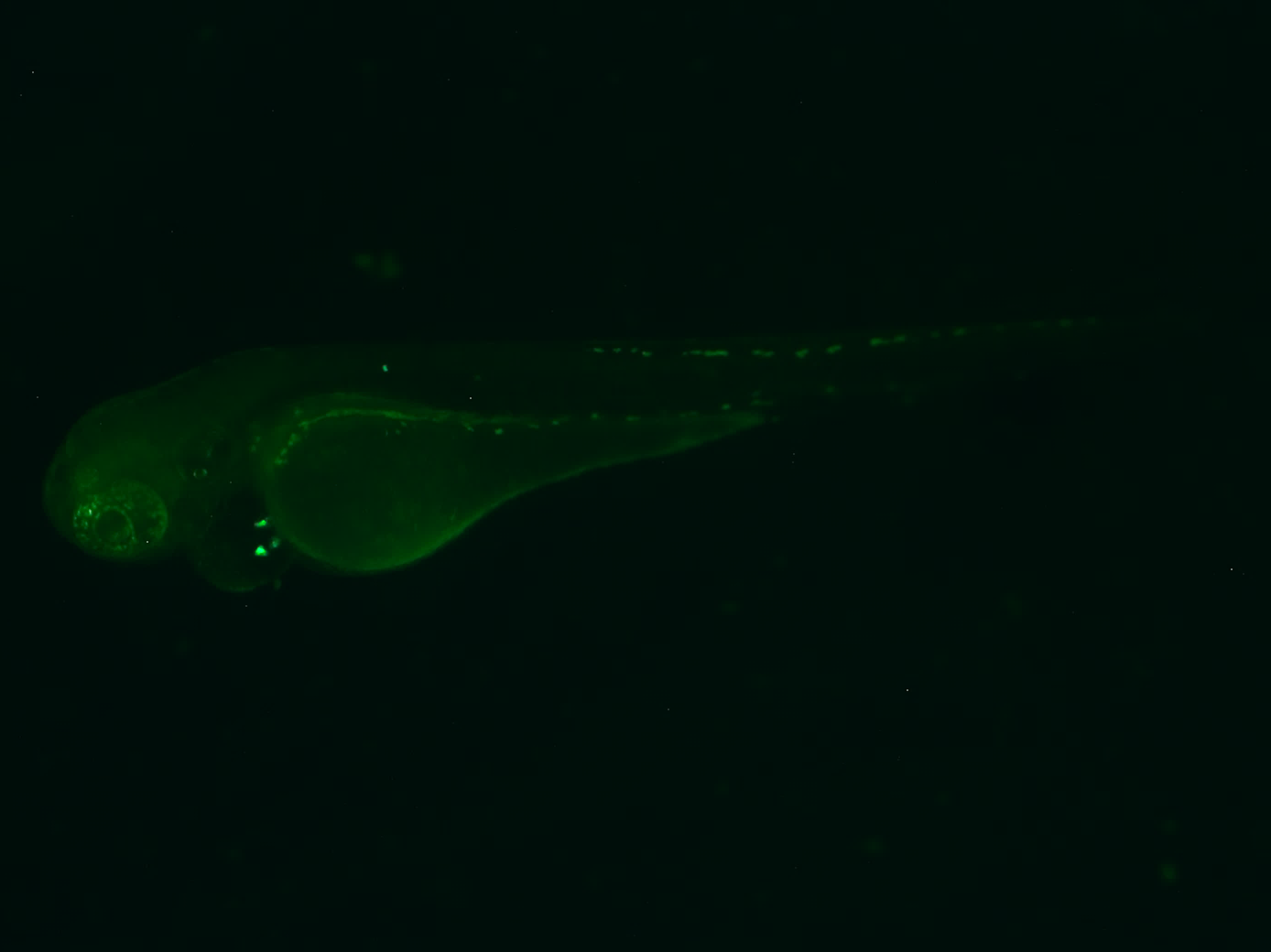

Supplement: Supplementary file 6 — Source data Fig. 4 [file 44319_2024_272_MOESM6_ESM.zip › Figure 4/4C/HUMAN SCAR-6.tif]

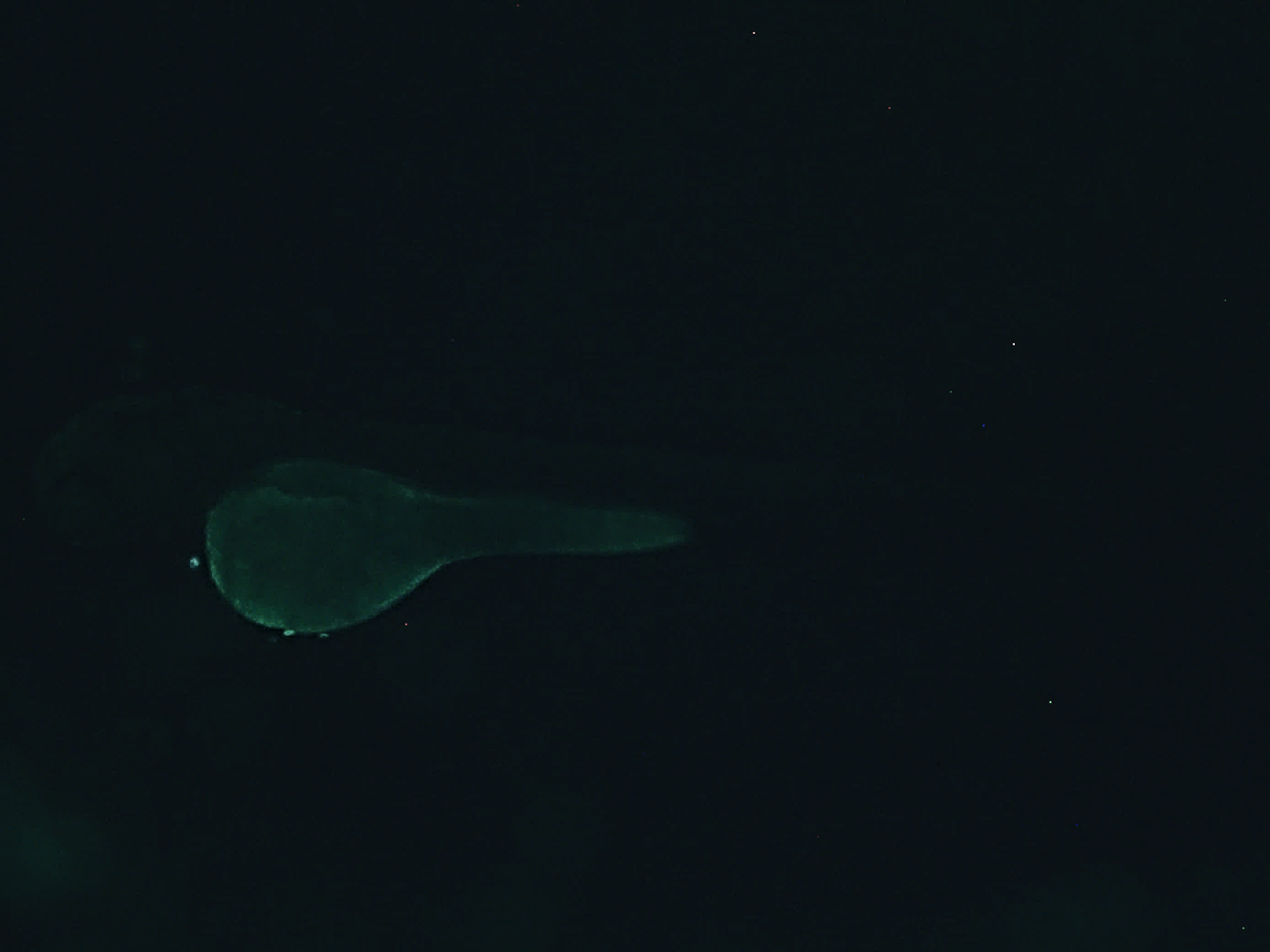

Supplement: Supplementary file 6 — Source data Fig. 4 [file 44319_2024_272_MOESM6_ESM.zip › Figure 4/4C/ZF SCAR-6.tif]

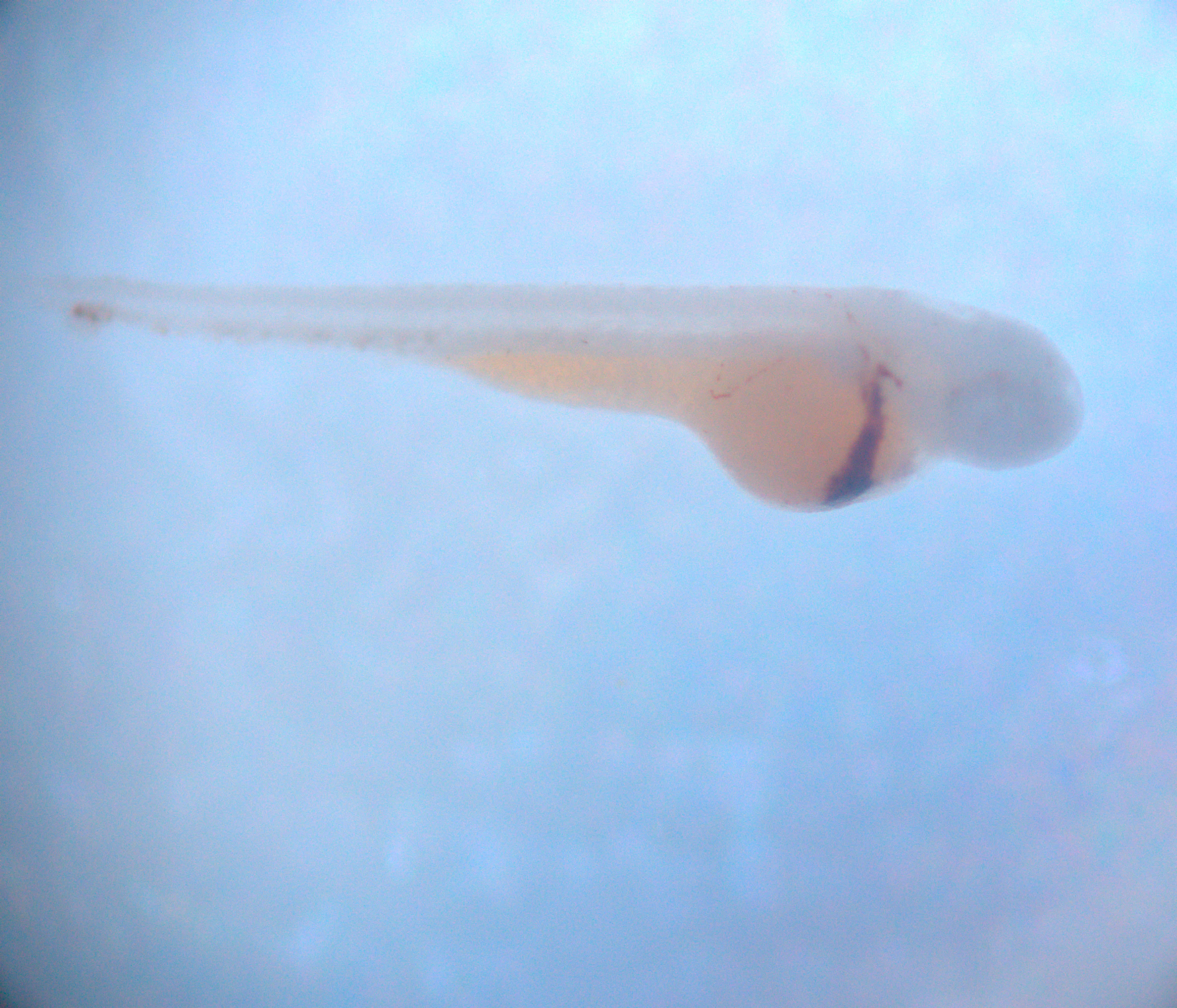

Supplement: Supplementary file 8 — Source data Fig. 6 [file 44319_2024_272_MOESM8_ESM.zip › Figure 6/6A/Control_BF1.tif]

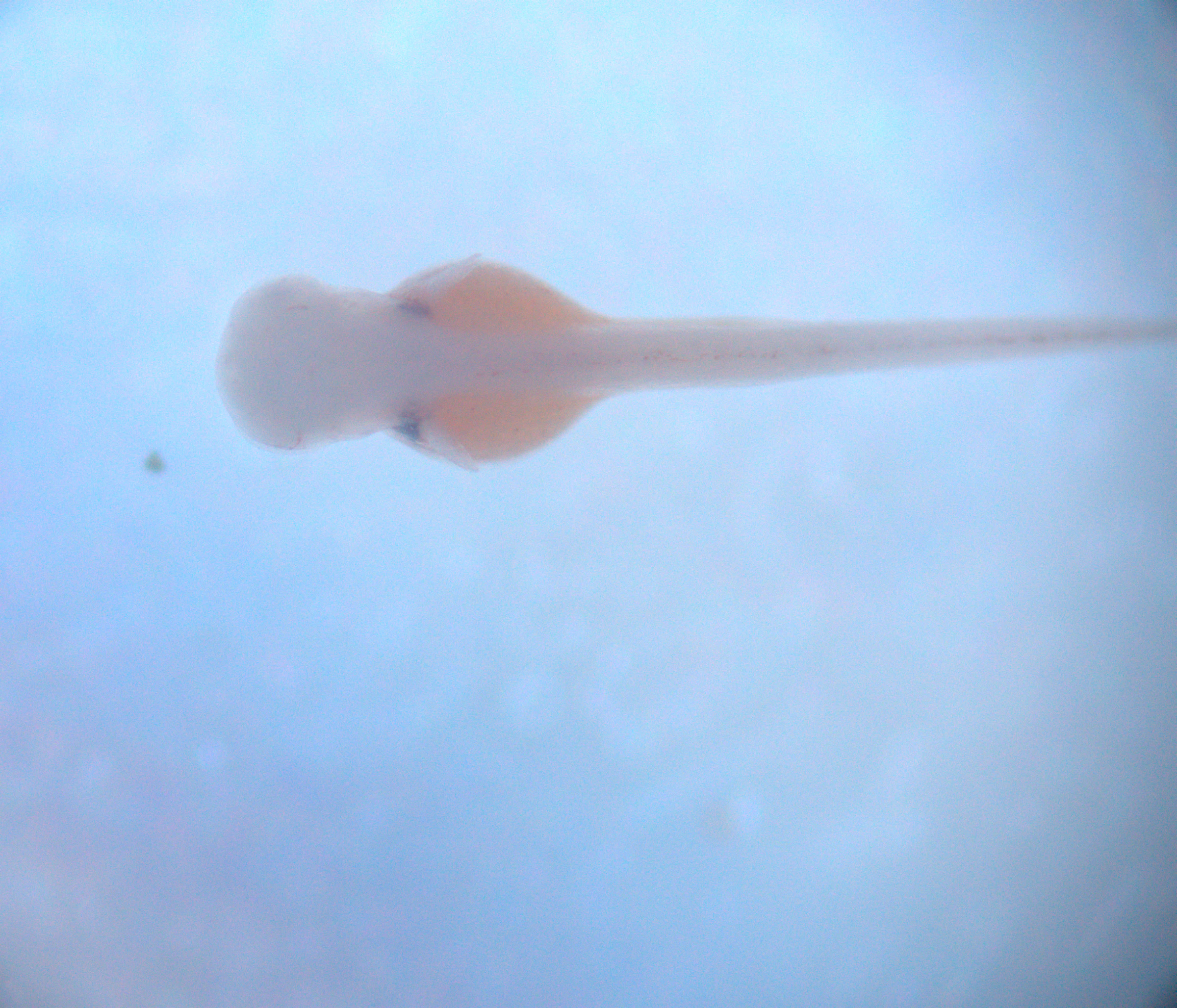

Supplement: Supplementary file 8 — Source data Fig. 6 [file 44319_2024_272_MOESM8_ESM.zip › Figure 6/6A/Control_BF2.tif]

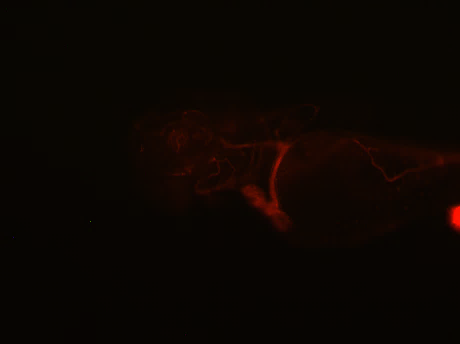

Supplement: Supplementary file 8 — Source data Fig. 6 [file 44319_2024_272_MOESM8_ESM.zip › Figure 6/6A/Control_dsRed.tif]

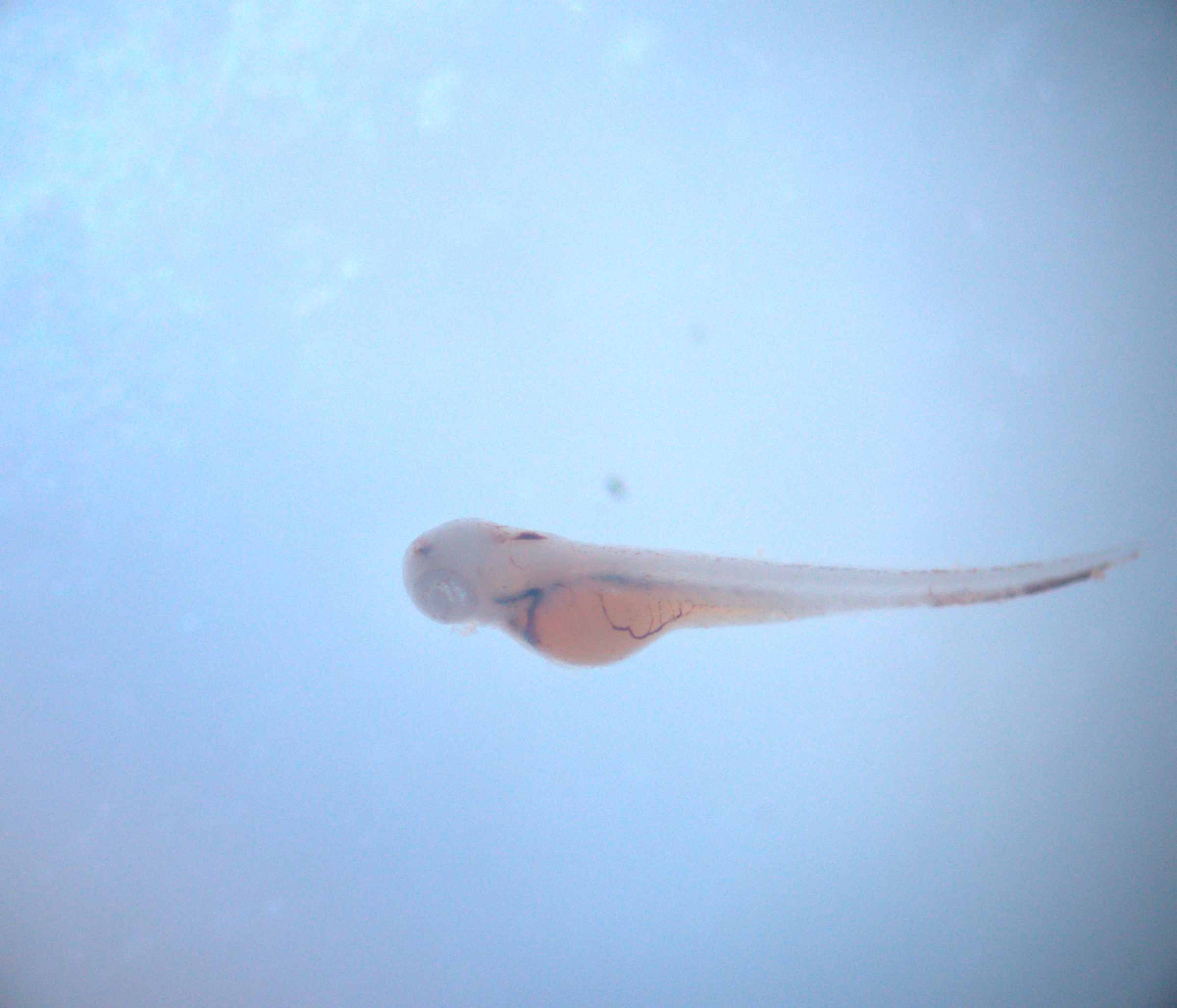

Supplement: Supplementary file 8 — Source data Fig. 6 [file 44319_2024_272_MOESM8_ESM.zip › Figure 6/6A/scar-6 del12 BF1.tif]

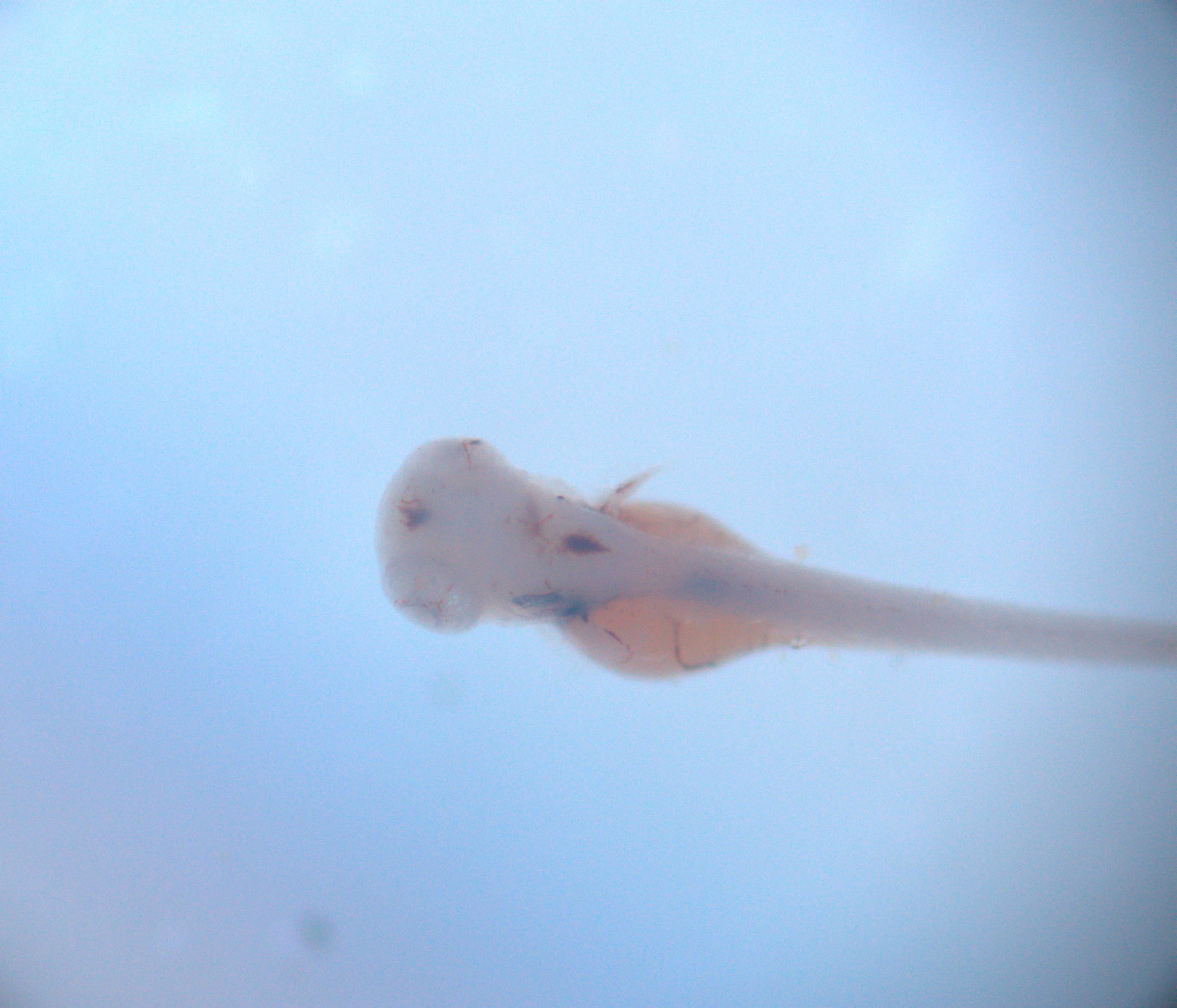

Supplement: Supplementary file 8 — Source data Fig. 6 [file 44319_2024_272_MOESM8_ESM.zip › Figure 6/6A/scar-6 del12 BF2.tif]

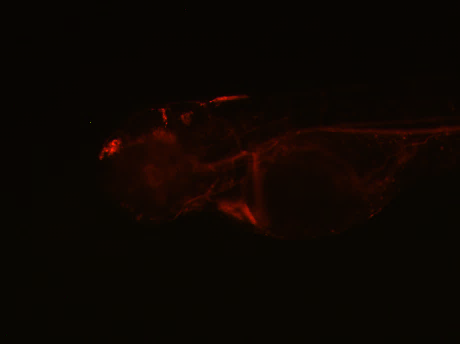

Supplement: Supplementary file 8 — Source data Fig. 6 [file 44319_2024_272_MOESM8_ESM.zip › Figure 6/6A/scar-6 del12 dsRed.tif]

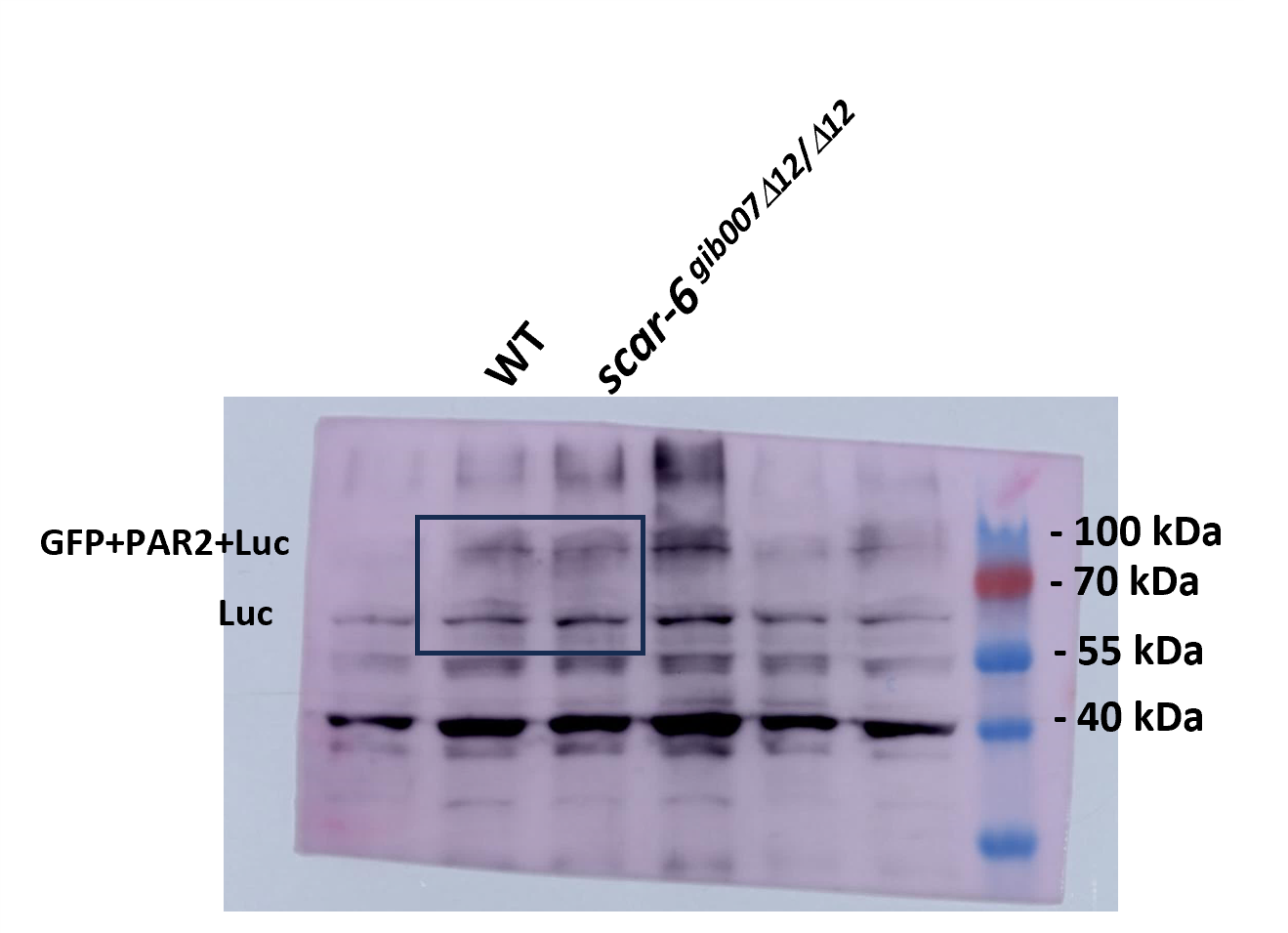

Supplement: Supplementary file 8 — Source data Fig. 6 [file 44319_2024_272_MOESM8_ESM.zip › Figure 6/6D/luciferase blot.tif]

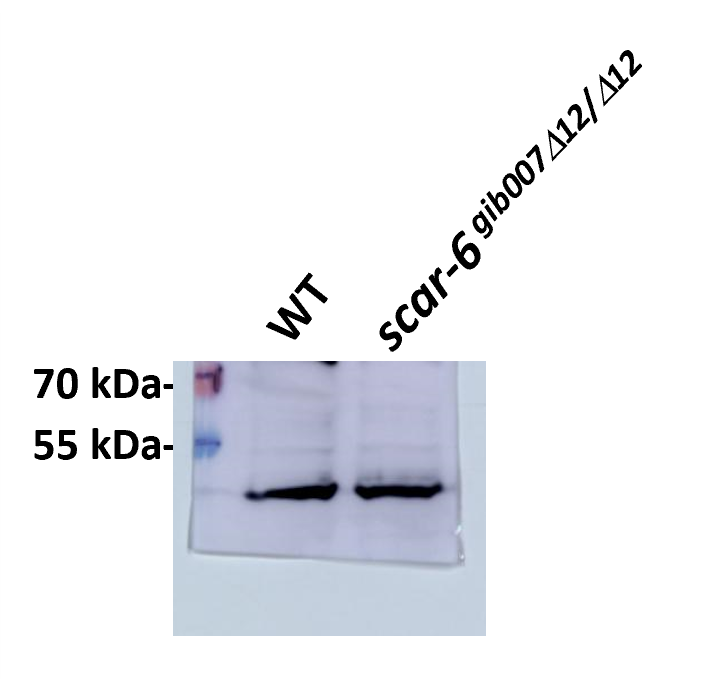

Supplement: Supplementary file 8 — Source data Fig. 6 [file 44319_2024_272_MOESM8_ESM.zip › Figure 6/6E/bactin BLOT.tif]

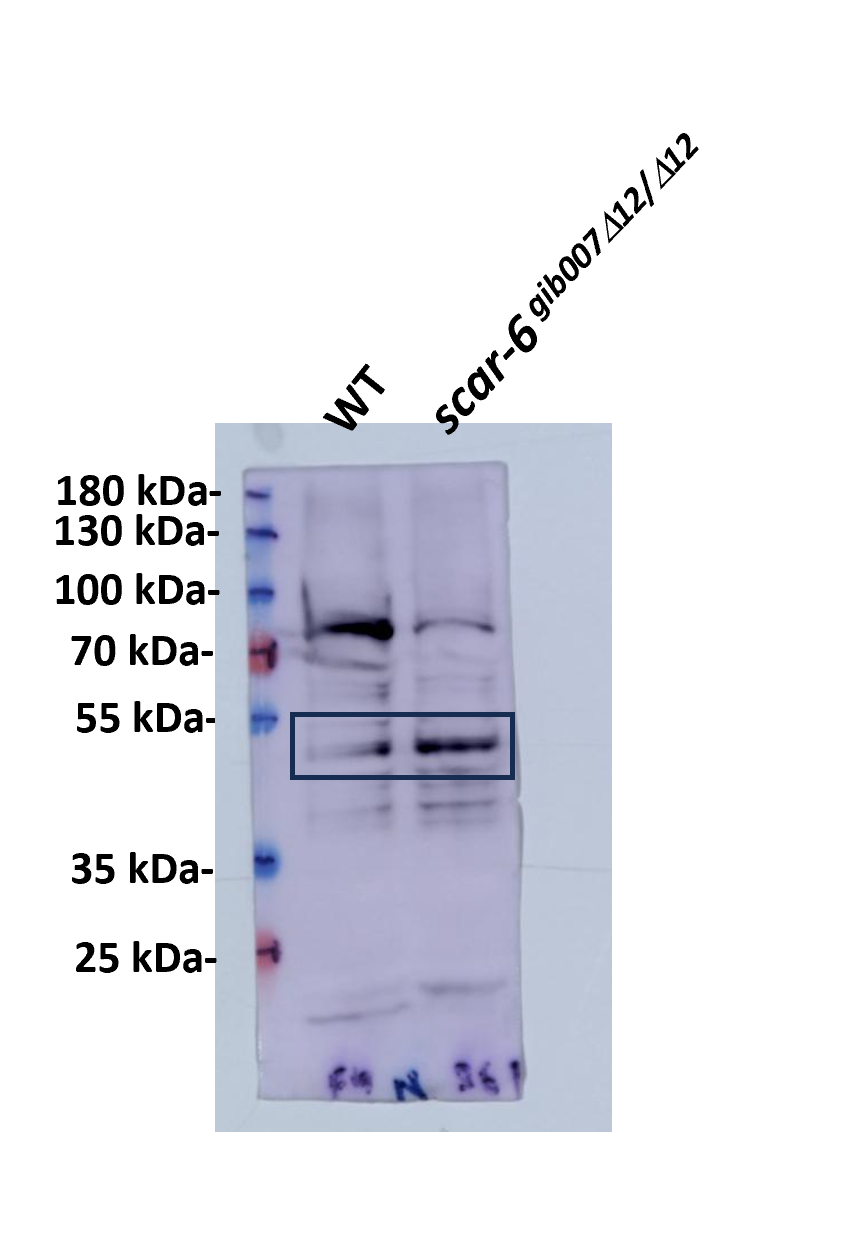

Supplement: Supplementary file 8 — Source data Fig. 6 [file 44319_2024_272_MOESM8_ESM.zip › Figure 6/6E/NfkB BLOT.tif]
