# Supplementary material for: SCAR-6 elncRNA locus epigenetically regulates PROZ and modulates coagulation and vascular function
Source: EMBO Rep. 2024 Oct 2;25(11):4950–78. doi: 10.1038/s44319-024-00272-w (PMC11549340; doi:10.1038/s44319-024-00272-w)
Supplement: Supplementary file 9 — Expanded View Figures [file 44319_2024_272_MOESM9_ESM.pdf]

Expanded View Figures

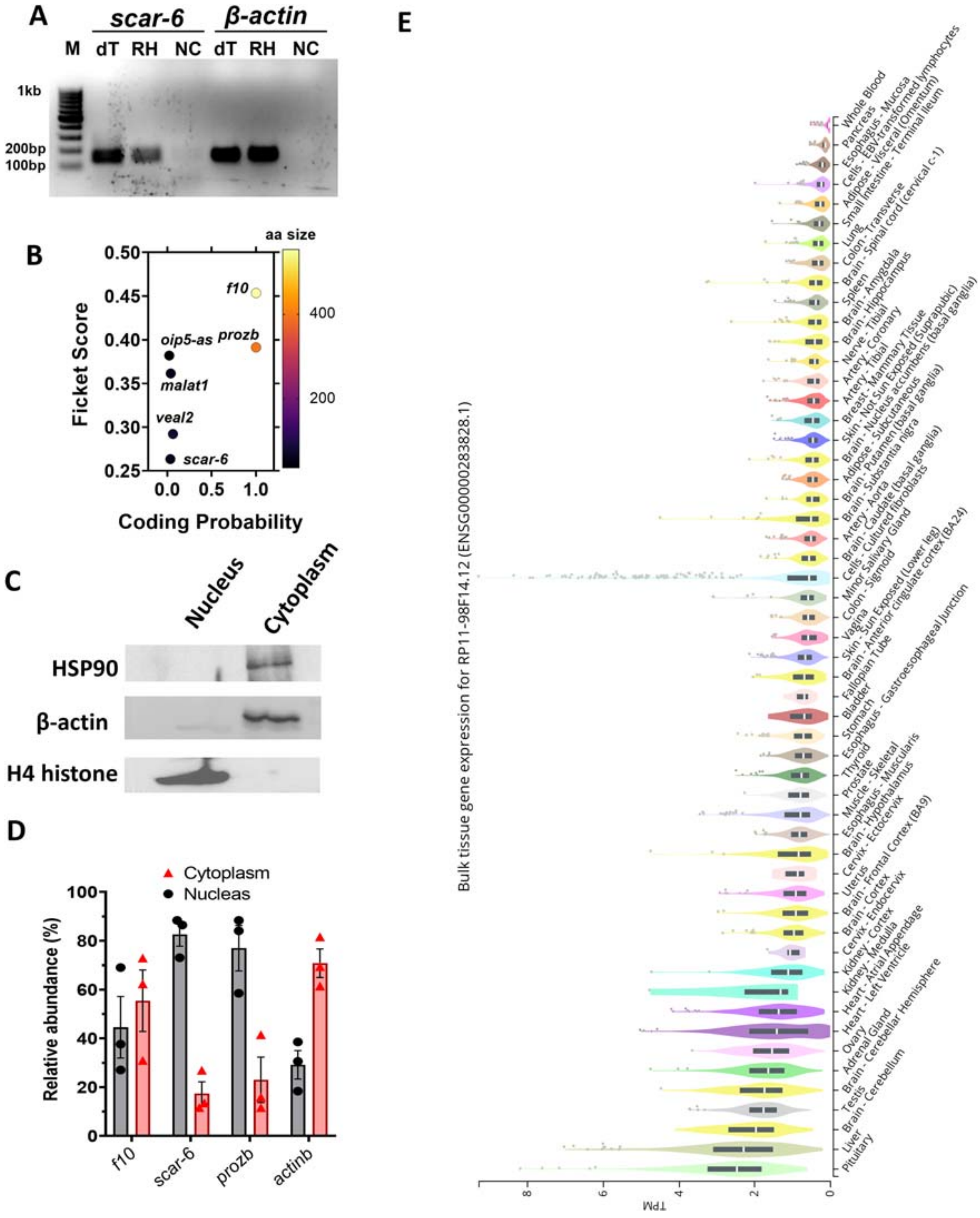

Figure EV1. ***scar-6* lncRNA transcript is ubiquitously expressed and nuclear enriched.**

(**A**) Image showing agarose gel electrophoresis of the PCR product derived from *scar-6* lncRNA and *β-actin*, amplified from cDNA synthesized using both oligo dT(dT) and random hexamer primers (RH). (**B**) Coding potentiality scores were calculated using CPC2 for *scar-6* and other lncRNA and protein-coding genes (*f10 and prozb*). (**C**) Western blot of *HSP90*, *β-actin* and H4 histone to confirm the purity of sub-cellular fractions of zebrafish cells. (**D**) The bar plot represents the relative abundance of the of *scar-6, f10* and *prozb* in different subcellular fractions quantified using qRT-PCR. The *scar-6* and *prozb* exhibit enrichment in the nucleus fraction and *f10* shows equal enrichment in cytoplasm and nucleus. Data from 3 different experiments were plotted as relative abundance percentages ± SEM. (**E**) The expression profile of human *SCAR-6* lncRNA across various tissues, as shown in the GTEx v8 database, is presented in transcripts per million (TPM). The box plot illustrates the data distribution, with the median indicated by a line inside each box, and the 25th and 75th percentiles represented by the lower and upper edges of the box, respectively. Outliers, defined as data points beyond 1.5 times the interquartile range, are also displayed. For sample sizes, please refer to the provided link: https://www.gtexportal.org/home/gene/ENSG00000283828.

© The Author(s)

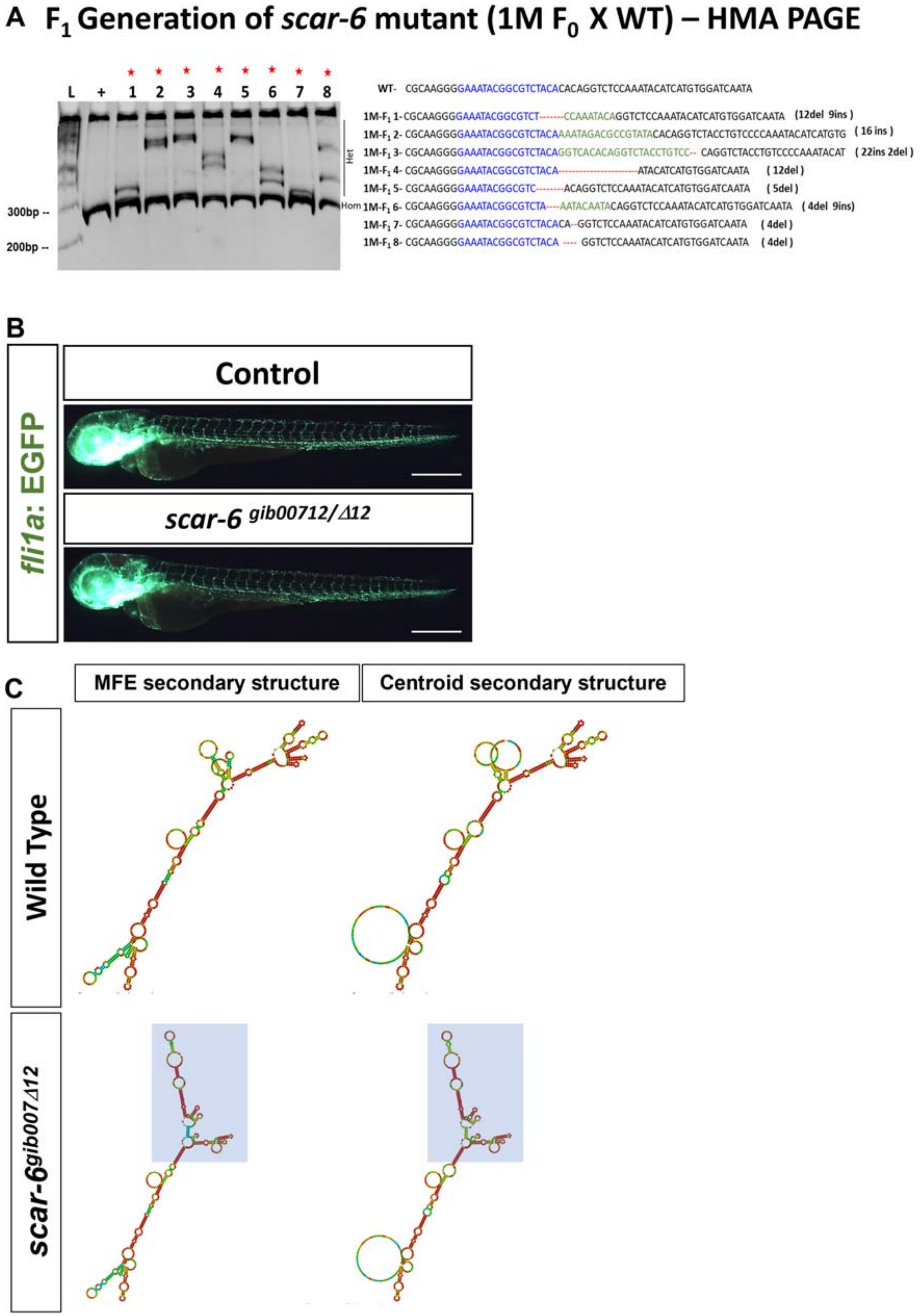
A F1 Generation of scar-6 mutant (1M F0 X WT) – HMA PAGE
L + 1 2 3 4 5 6 7 8
300bp --
200bp --
Het
Hom
WT- CGCAAGGGGAAATACGGCGTCTACACACAGGTCTCCAAATACATCATGTGGATCAATA
1M-F1 1- CGCAAGGGGAAATACGGCGTCT-------CCAAATACAGGTCTCCAAATACATCATGTGGATCAATA (12del 9ins)
1M-F1 2- CGCAAGGGGAAATACGGCGTCTACAAAATAGACGCCGTATACACAGGTCTACCTGTCCCCAAATACATCATGTG (16 ins)
1M-F1 3- CGCAAGGGGAAATACGGCGTCTACAGGTCACACAGGTCTACCTGTCC-- CAGGTCTACCTGTCCCCAAATACAT (22ins 2del)
1M-F1 4- CGCAAGGGGAAATACGGCGTCTACA------------ATACATCATGTGGATCAATA (12del)
1M-F1 5- CGCAAGGGGAAATACGGCGTC-----ACAGGTCTCCAAATACATCATGTGGATCAATA (5del)
1M-F1 6- CGCAAGGGGAAATACGGCGTCTA----AATACAATACAGGTCTCCAAATACATCATGTGGATCAATA (4del 9ins)
1M-F1 7- CGCAAGGGGAAATACGGCGTCTACACA--GGTCTCCAAATACATCATGTGGATCAATA (4del)
1M-F1 8- CGCAAGGGGAAATACGGCGTCTACA ---- GGTCTCCAAATACATCATGTGGATCAATA (4del)
B
Control
fli1a: EGFP
scar-6 gib00712/Δ12
C
MFE secondary structure
Centroid secondary structure
Wild Type
scar-6gib007Δ12

Figure EV2. CRISPR-Cas9 mediated mutant generation of *scar-6* lncRNA gene.

(**A**) DNA-PAGE gel illustrating the results of the heteroduplex mobility assay (HMA) conducted on the *scar-6* targeted region in F_1 *scar-6* mutant adult zebrafish, with genotypes identified by in-dels. Extra bands in the PAGE gel represent heteroduplex templates formed due to heterozygosity in the target region. The red asterisk denotes heterozygous mutant animals. (**B**) Representative image showing blood vessels of 3 dpf zebrafish progeny derived from wild-type gib004Tg(*fli1a*:EGFP;*gata1a*:DsRed) and *scar-6*$^{gib007\Delta12/\Delta12}$ zebrafish. 2.5× magnification; scale bar = 500 µm. (**C**) Representative image showing secondary structure of scar-6 RNA under wildtype and 12-bp edited condition. The highlighted region represent change in the structure of lncRNA.

 © The Author(s)

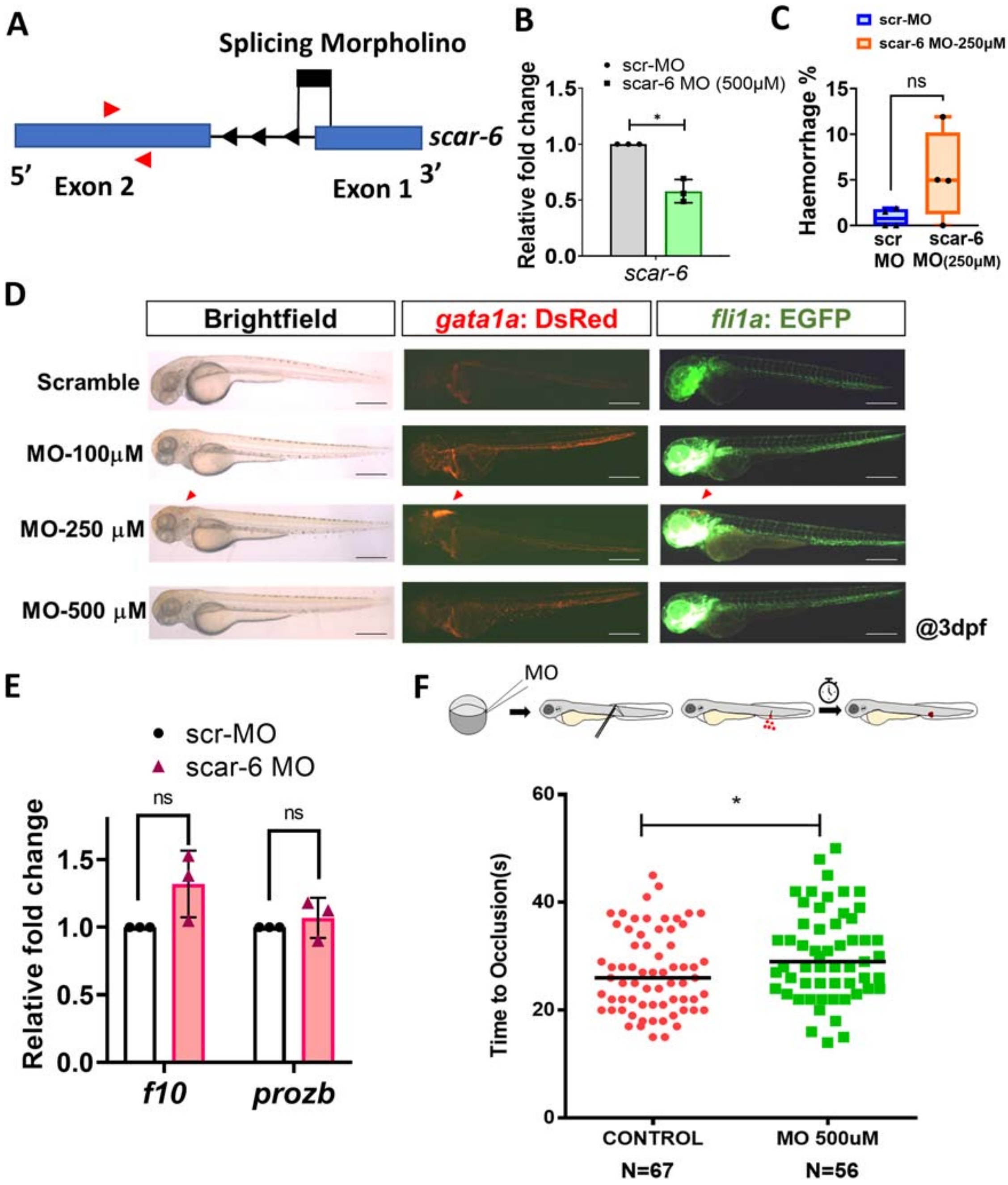

Figure EV3. Knockdown of *scar-6* shows no significant phenotype.

(**A**) Schematic of antisense splice blocking morpholino oligo targeting the exon 1 splicing junction of *scar-6* lncRNA. (**B**) Relative fold change expression of *scar-6* upon of knockdown with splice blocking morpholino in zebrafish at 3 dpf. Data from 3 independent biological replicates plotted as mean fold change ± standard deviation; **P* < 0.05 (two-tailed unpaired t-test). (**C**) Box and whisker plot representing the percentage of animals with haemorrhage phenotype in zebrafish embryos injected with 250 μM of morpholino. Data from 4 independent experiments are represented by boxes indicating the interquartile range (25th to 75th percentiles), with the horizontal line within each box denoting the median. Whiskers extend to 1.5 times the interquartile range to define the minimum and maximum values, while individual points represent data from each experiment.: ns, not significant (two-tailed unpaired t-test). (**D**) Representative image of gib004Tg(*fli1a*:EGFP;*gata1a*:DsRed) zebrafish injected with different concentration of morpholino at 3 dpf. Red arrowhead denotes hemorrhage in the animals. 2.5× magnification, scale bar = 500 μm. (**E**) Relative fold change expression of *f10* and *prozb* upon knockdown of *scar-6* lncRNA with splice blocking morpholino in zebrafish. Data from 3 independent biological replicates plotted as mean fold change ± standard deviation; ns, not significant (two-tailed unpaired t-test). (**F**) Coagulation assay plot calculating the time of occlusion (s) in different individual zebrafish in control and 500uM morpholino injected zebrafish. Each point represents the occlusion time of individual zebrafish segregated based on genotype; **P* < 0.05 (Mann-Whitney U).

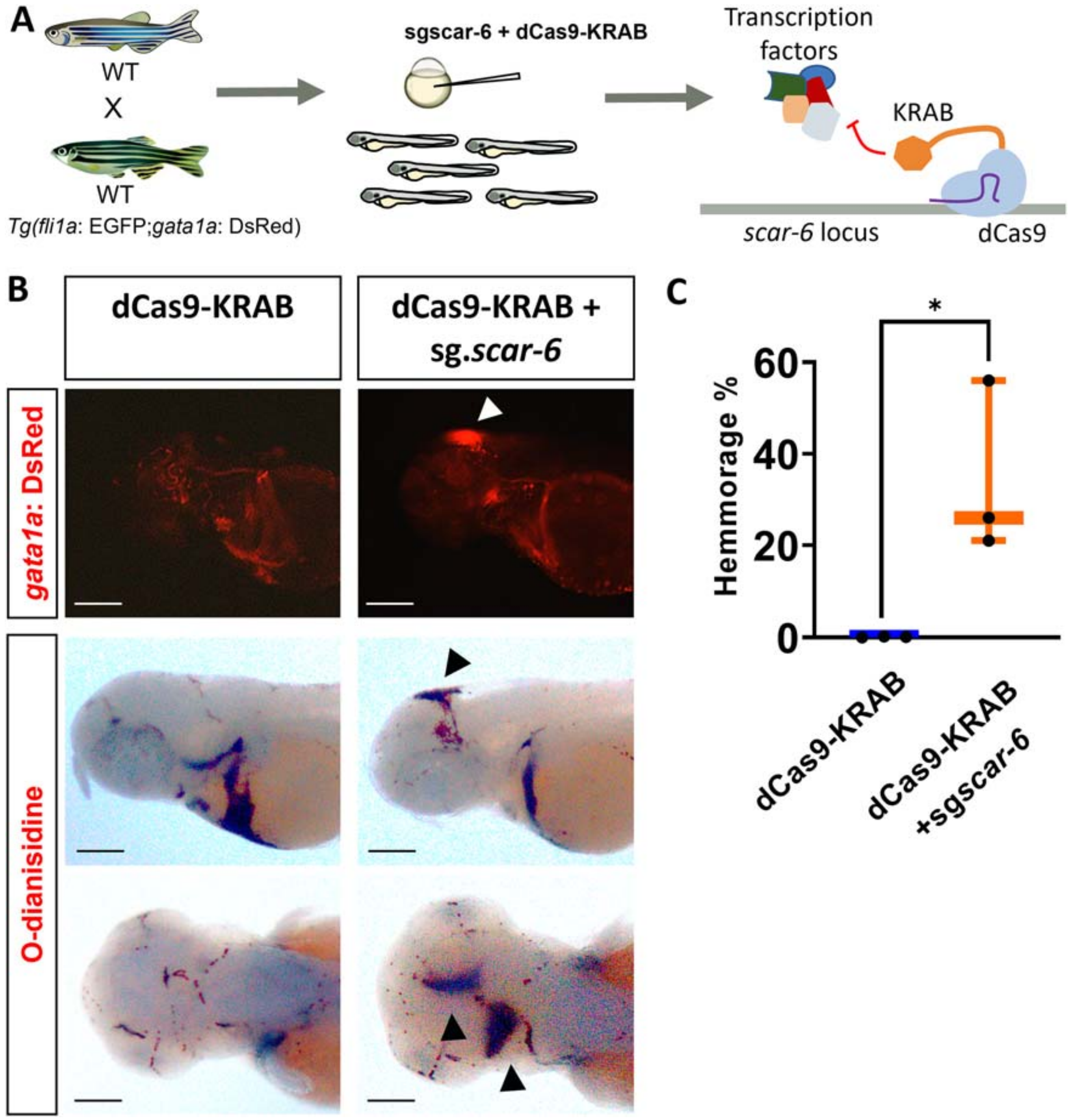

Figure EV4. CRISPRi of *scar-6* locus exhibits haemorrhage phenotype.

(**A**) Representation of CRISPR-dCas9-KRAB mediated inhibition of TF binding on *scar-6* locus. (**B**) Representative image showing fluorescence for blood (*gata1a:DsRed)* in the cranial region of 3 dpf zebrafish and o-dianisidine stating of RBC blood cells in control and dCas-9-KRAB + sg*scar-6* injected zebrafish. The black and white arrowhead denotes hemorrhage in the animals. 4× magnification; scale bar = 200 µm. (**C**) Box and whisker plot representing the percentage of animals exhibiting haemorrhage phenotype in control and dCas-9-KRAB + sg*scar-6* injected zebrafish. Data from 3 independent biological replicates are represented by boxes indicating the interquartile range (25th to 75th percentiles), with the horizontal line within each box denoting the median. Whiskers extend to 1.5 times the interquartile range to define the minimum and maximum values, while individual points represent data from each experiment; *$p < 0.05$ (two-tailed unpaired t-test).

 © The Author(s)

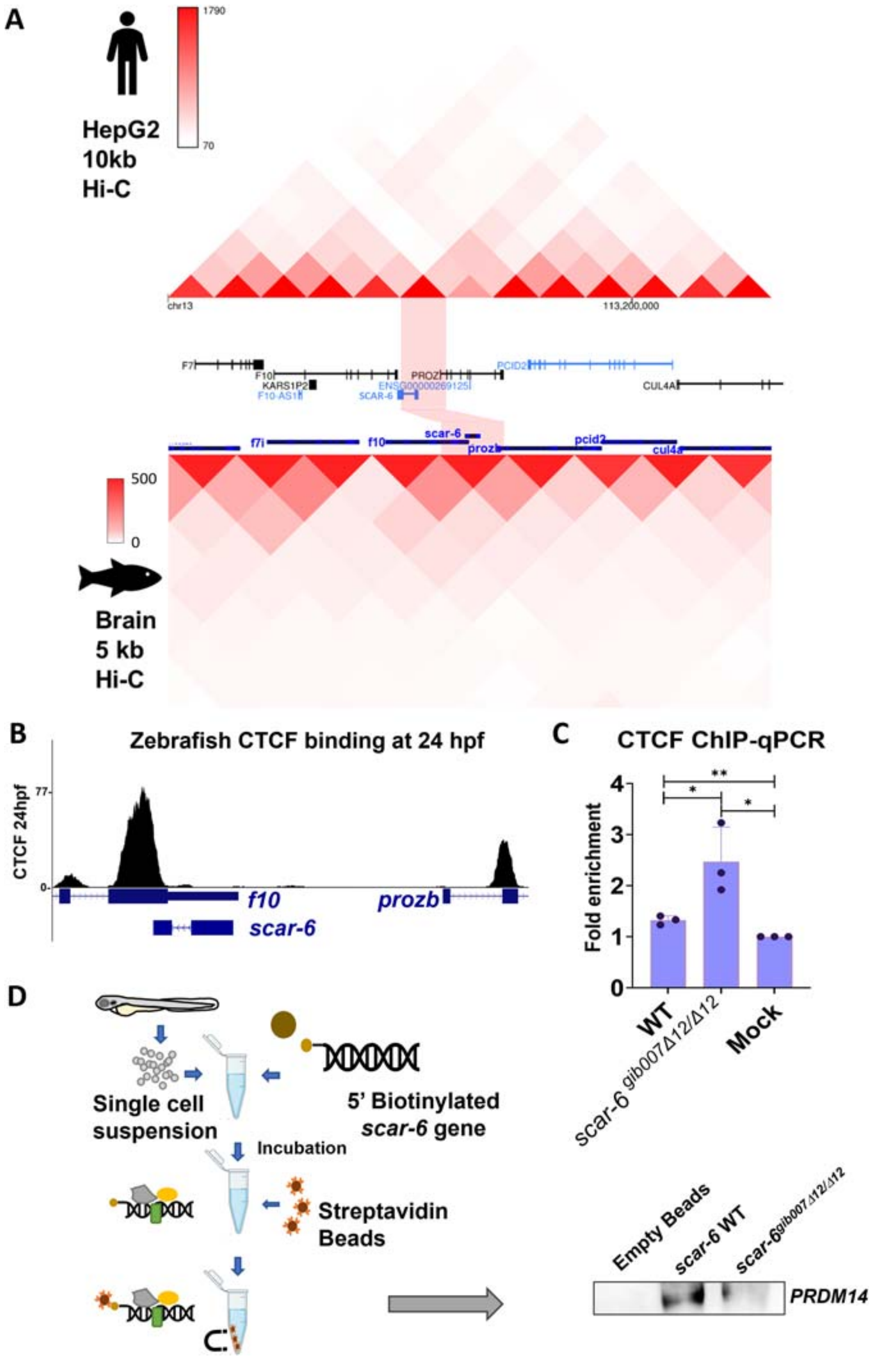

Figure EV5. Sub-TAD looping mediated enhancer-promoter interaction of *scar-6/prozb* locus.

(**A**) Hi-C heatmap representation of Human *SCAR-6* locus in HepG2 at 10 kb resolution from ENCODE database (ENCODE Project Consortium et al, 2020; Wang et al, 2018) and zebrafish *scar-6* locus in brain tissue at 5 kb resolution from (Yang et al, 2020). (**B**) UCSC genome browser snapshot of data for CTCF binding peaks at 24 hpf of zebrafish for at *scar-6* and *prozb* locus (Pérez-Rico et al, 2020). (**C**) Bar plot representing ChIP-qPCR quantifying fold enrichment using CTCF antibody for *scar-6* locus in wild type and *scar-6*$^{gib007\Delta12/\Delta12}$ mutant zebrafish. Data from 3 independent biological replicates plotted as mean fold enrichment ± standard deviation; *$P < 0.05$, **$P < 0.01$ (two-tailed unpaired t-test). (**D**) Schematic and western blot of DNA-pulldown assay was performed using streptavidin tagged scar-6 gene DNA in zebrafish, and immuno blotting was done using *prdm14* antibody. $N = 3$.
